# GSTM1 suppresses cardiac fibrosis post-myocardial infarction through inhibiting lipid peroxidation and ferroptosis

**DOI:** 10.1186/s40779-025-00610-6

**Published:** 2025-05-31

**Authors:** Kai-Jie Chen, Yue Zhang, Xin-Yi Zhu, Shuo Yu, Yao Xie, Cheng-Jiang Jin, Yi-Min Shen, Si-Yu Zhou, Xiao-Ce Dai, Sheng-An Su, Lan Xie, Zheng-Xing Huang, Hui Gong, Mei-Xiang Xiang, Hong Ma

**Affiliations:** 1 Department of Cardiology, The Second Affiliated Hospital, School of Medicine, State Key Laboratory of Transvascular Implantation Devices, Cardiovascular Key Laboratory of Zhejiang Province, Zhejiang University, Hangzhou, 310009 China; 2 Department of Anesthesiology, The Second Affiliated Hospital, School of Medicine, Zhejiang University, Hangzhou, 310009 China; 3 College of Computer Science and Technology, Zhejiang University, Hangzhou, 310009 China; 4 Shanghai Institute of Cardiovascular Diseases, Zhongshan Hospital, Fudan University, Shanghai, 200032 China

**Keywords:** GSTM1, Ferroptosis, Cardiac fibrosis, Myocardial infarction (MI), Lipid peroxidation, Glutathione peroxidase 4, Reactive oxygen species (ROS)

## Abstract

**Background:**

Cardiac fibrosis following myocardial infarction (MI) drives adverse ventricular remodeling and heart failure, with cardiac fibroblasts (CFs) playing a central role. GSTM1 is an important member of the glutathione S-transferase (GSTs) family, which plays an important role in maintaining cell homeostasis and detoxification. This study investigated the role and mechanism of GSTM1 in post-MI fibrosis.

**Methods:**

Multi-omics approaches (proteomics/scRNA-seq) identified GSTM1 as a dysregulated target in post-MI fibroblasts. Using a murine coronary ligation model, we assessed GSTM1 dynamics via molecular profiling, such as Western blotting, immunofluorescence, and real-time quantitative polymerase chain reaction. AAV9-mediated cardiac-specific GSTM1 overexpression was achieved through systemic delivery. In vitro studies employed transforming growth factor-β (TGF-β)-stimulated primary fibroblasts with siRNA/plasmid interventions. Mechanistic insights were derived from transcriptomics and lipid peroxidation assays.

**Results:**

The expression of GSTM1 in mouse CFs after MI was significantly down-regulated at both transcriptional and protein levels. In human dilated cardiomyopathy (DCM) patients with severe heart failure, GSTM1 expression was decreased alongside aggravated fibrosis. Overexpression of GSTM1 in post-MI mice improved cardiac function, while significantly reducing infarct size and fibrosis compared with the control group. In vitro models demonstrated that GSTM1 markedly attenuated collagen secretion and activation of fibroblasts, as well as suppressed their proliferation and migration. Further studies revealed that GSTM1 overexpression significantly inhibited the generation of intracellular and mitochondrial reactive oxygen species (ROS) under pathological conditions, suggesting that GSTM1 exerts an antioxidative stress effect in post-infarction fibroblasts. Further investigation of molecular mechanisms indicated that GSTM1 may suppress the initiation and progression of fibrosis by modulating lipid metabolism and ferroptosis-related pathways. Overexpression of GSTM1 significantly reduced lipid peroxidation and free ferrous iron levels in fibroblasts and mitochondria, markedly decreased ferroptosis-related indicators, and alleviated oxidative lipid levels [such as 12-hydroxyeicosapentaenoic acid (HEPE) and 9-, 10-dihydroxy octadecenoic acid (DHOME)] under fibrotic conditions. GSTM1 enhanced the phosphorylation of STAT3, thereby upregulating the downstream expression of glutathione peroxidase 4 (GPX4), reducing ROS production, and mitigating fibroblast activation and phenotypic transformation by inhibiting lipid peroxidation.

**Conclusions:**

This study identifies GSTM1 as a key inhibitor of fibroblast activation and cardiac fibrosis, highlighting its ability to target ferroptosis through redox regulation. AAV-mediated GSTM1 therapy demonstrates significant therapeutic potential for improving outcomes post-MI.

**Supplementary Information:**

The online version contains supplementary material available at 10.1186/s40779-025-00610-6.

## Background

Cardiovascular disease, with myocardial infarction (MI) as a leading cause, remains a significant global health challenge [1–3]. The loss of cardiomyocytes and subsequent excessive fibrosis often results in the development of heart failure. In this pathological process, cardiac fibroblasts (CFs) play a pivotal role in causing abnormal accumulation of extracellular matrix (ECM) and thereafter cardiac dysfunction [4, 5]. While initially beneficial for tissue repair, over-activated fibroblasts can lead to adverse ventricular remodeling, decreased ventricular compliance, reduced cardiac function, and ultimately heart failure [6]. Thus, it remains a pressing clinical challenge to find ways to prevent adverse ventricular remodeling and delay heart failure.

Reactive oxygen species (ROS) have gained significant momentum in cardiovascular research [7, 8] due to their critical role in regulating various cellular processes, including signal transduction, inflammation, apoptosis, and fibroblast transformation [9]. These processes collectively contribute to the development of cardiac fibrosis, a pathological hallmark of MI. Studies have revealed that ROS regulates cardiac fibrosis through multiple pathways, such as the Wnt/β-catenin, nuclear factor kappa-B (NF-κB), and signal transducer and activator of transcription 3 (STAT3) signaling cascades [10, 11]. Additionally, ROS can modulate mitochondrial function and calcium ion homeostasis, further impacting cardiac fibrosis [12, 13]. Given ROS’s pivotal role in cardiac fibrosis, targeting ROS clearance has emerged as a promising therapeutic strategy [14]. Antioxidants and specific medications designed to reduce excessive ROS hold the potential to alleviate or reverse the progression of this condition. However, currently, there are no clinically approved drugs or studies demonstrating a significant intervention effect on post-MI fibrosis. A deeper understanding of the mechanisms by which ROS regulates cardiac fibrosis is essential for developing novel therapeutic strategies and drugs. This knowledge could potentially lead to breakthroughs in treating cardiovascular diseases characterized by cardiac fibrosis.

Glutathione S-transferase (GST) is a family of multifunctional enzymes found in various organisms [15]. Their primary role is to catalyze the conjugation of electrophilic compounds, both endogenous and exogenous, with reduced glutathione [16], thereby facilitating detoxification. Among the GST subtypes, GSTM1 is one of the most prevalent. GSTM1 effectively neutralizes free radicals by transferring glutathione, protecting cells from oxidative damage caused by peroxidation [17].

This study investigated the role of GSTM1 in post-MI fibrosis and its regulation of lipid peroxidation. By elucidating GSTM1’s function and mechanisms, we aimed to understand how it inhibits iron-mediated oxidative stress and lipid peroxidation, thereby mitigating fibrosis development. These findings may inform the development of novel therapeutic strategies to improve outcomes following MI.

## Methods

### Human tissue

Three non-disease human hearts came from three healthy donors who died of brain stem bleeding, and three hearts with heart failure came from end-stage dilated cardiomyopathy (DCM) patients. All subjects are fully informed, with written consent from the patient or their relatives. All human studies were approved by the Human Research Ethics Committee of the Second Affiliated Hospital of Zhejiang University School of Medicine (2014-160) and were conducted following the principles of the Declaration of Helsinki.

### Animal experiment

A total of 36 C57BL/6J male mice and 18 female mice 6–10 weeks, were used in this study. All animals were housed in a facility adhering to the guidelines of the Zhejiang University Animal Care and Use Committee. Experimental procedures were conducted in accordance with approved protocols.

Mice received a single tail vein injection of 1 × 10^12^ vg/ml, 100 μl per mouse adeno-associated virus serotype 9 (AAV9) virus (negative control or GSTM1 overexpression) 2 weeks before MI surgery [18]. Baseline cardiac function was assessed using echocardiography before the procedure. A previously described mouse model of MI was employed [19]. Briefly, mice were anesthetized with intraperitoneal pentobarbital sodium (50 mg/kg) and underwent left anterior descending coronary artery ligation using a 7 − 0 prolene suture. Sham surgery involved the same procedure without ligation. Each group comprised at least 6 mice. Ejection fraction was measured on the third post-MI day to confirm successful model establishment.

At the end of the experiment, the mice were euthanized by intravenous injection of a lethal dose of pentobarbital sodium (100 mg/kg). All procedures involving animals have been approved by the Zhejiang University Animal Care and Utilization Committee (2022-113) and are in accordance with the Council of the European Communities Directive 2010/63/EU on the protection of animals used for experimental purposes.

### Isolation of CFs

CFs were isolated from male C57BL/6J mice within the age range of 6–8 weeks. The hearts of adult mice were excised, followed by the removal of the atria. The remaining cardiac tissue was minced into small pieces. Digestion was carried out at 37 °C, utilizing a combination of trypsin (Gibco, United States) and collagenase type II (Invitrogen, United States). After digestion, the cells were subjected to centrifugation, and the resulting pellet was resuspended. The cells were then seeded, and after 1.5 h, the culture medium was replaced. At this specific time point, the adherent cells primarily comprised CFs. All animal experiments were conducted with the approval of the Zhejiang University Second Affiliated Hospital Animal Experiment Ethics Committee (approval date: July 28, 2022, Ref. 2022-113).

### Western blotting

Cells and tissues were lysed in RIPA buffer (beyotime, China) containing proteinase inhibitor and phosphatase inhibitor (Thermo Fisher, United States) to extract total proteins and analyzed as previously described [20]. The BCA protein assay kit (Thermo Fisher, United States) was employed to quantify the protein concentration of each protein sample. Subsequently, the protein samples were separated by SDS-PAGE and transferred onto PVDF membranes. These membranes were then blocked with 5% skim milk in PBST for a duration of 1 h at ambient temperature. Primary antibodies, as specified in the accompanying table, were added to the membranes, followed by overnight incubation at 4 °C. After a thorough washing process, horseradish peroxidase (HRP)-conjugated IgG was utilized as the secondary antibody. The resulting antigen–antibody complexes were visualized through the application of an ECL system (Amersham ImageQuant 800, United Kingdom).

### Real-time quantitative polymerase chain reaction (RT-qPCR)

Total RNA extraction was conducted using TRIzol reagent (Thermo Fisher, United States), adhering to the manufacturer’s established protocols [20]. The extracted RNA was subsequently reverse-transcribed into cDNA utilizing the RprimerScript RT Reagent Kit (TaKaRa, Japan). RT-qPCR was executed on a Lightcycler 480 II instrument (Roche, Switzerland), employing TB Green Premix Ex Taq (TaKaRa, Japan). β-actin served as an internal reference for the normalization of mRNA expression levels. The primer sequences of all genes are detailed in the Additional file 1: Table S1.

### Histological analysis

The excised heart tissue was immersed in a 4% paraformaldehyde solution (Biosharp, China) and subsequently embedded in paraffin and cut longitudinally into 4 μm thick cross-sectional slices Hematoxylin and eosin (HE), Masson’s trichrome, and Sirius red staining were conducted to analyze tissue morphology, evaluate tissue fibrosis, and assess collagen content. Each heart was selected for 5 different longitudinal position slices for comprehensive statistical analysis [20] (Additional file 1: Fig. S1).

HE: the sections that have been injected with distilled water are placed in an aqueous hematoxylin solution and stained for 5 min. Acid water and ammonia water color separation, 30 s each. Rinse under running water for 1 h and then enter distilled water for 30 s Dehydrate in 70% and 90% alcohol for 10 min each. Alcohol eosin stain solution for 2–3 min. The stained sections were dehydrated by pure alcohol and then made transparent by xylene. Drop the transparent slice with Canadian gum and cover it with a cover glass to seal.

Masson’s trichrome: the paraffin sections prepared before were placed in the oven at 68 °C to bake the slices, and then dewaxed to water. Slice into Bouin solution, overnight at 37 °C, rinse slices with running water until the yellow color on the slices disappears. The sections were stained with ponceau fuchsin drops for 10 min and rinsed slightly with distilled water.

The sections were stained with the phosphomolybdic acid solution for about 10 min, the upper solution was poured away, and the sections were directly stained with aniline blue dyeing solution for 5 min without washing, and treated with the weak acid solution for 2 min. Then slice into 95% ethanol for rapid dehydration, anhydrous ethanol dehydration 3 times, each time for 5–10 s. Seal the sheet with a neutral resin solution containing xylene and air dry in the fume hood. The sections were transferred to the optical microscope for observation and the whole heart image was taken. The blue is collagen fibers and the red is normal heart muscle tissue.

Sirius red: the paraffin sections were placed in an oven at 68 °C and then dewaxed to water. The dye droplets were placed on the surface of the section and incubated at room temperature (25 °C) for 1 h. Gently rinse slices with water 2–3 times. Wash the slices with anhydrous ethanol 2–3 times, seal the slices with a neutral resin solution containing xylene, and air dry in the fume hood. The sections were transferred to an ordinary optical microscope for observation, and the whole heart image was taken. The red is collagen fibers and the yellow is normal cardiac tissue.

### Immunofluorescence staining

Frozen heart sections were embedded in OCT compound (SAKURA, Japan) and subsequently sectioned. The resulting 4-μm-thick frozen sections were fixed in a 15% paraformaldehyde PBS solution for 15 min. Subsequently, the sections were permeabilized with a 0.5% Triton X-100 (Sigma-Aldrich, United States) PBS solution for 15 min. After blocking with 10% donkey serum in PBS for 1 h at ambient temperature, the sections were incubated with primary antibodies overnight at 4 °C. Following a thorough washing process, the sections were incubated with secondary antibodies for 1 h at ambient temperature, followed by DAPI counterstaining. Ultimately, the slides were imaged using a fluorescence microscope (Leica, Germany). At least 100 cells were measured from different fields of view of each group of different samples for statistical analysis.

### Statistical analysis

The data were presented as means with the standard error of the mean derived from at least 3 independent experiments. The normal distribution of variables was confirmed through the Kolmogorov-Smirnov test and Q-Q plots analysis. Statistical differences between two groups were determined using the Student’s *t*-test, while comparisons among three or more groups were conducted using ANOVA followed by Bonferroni multiple comparison tests. For non-normally distributed data, multiple comparisons were performed using the Kruskal-Wallis test. A *P*-value of less than 0.05 was considered statistically significant. Statistical calculations were carried out using GraphPad Prism 8.0 or SPSS version 20.0.

## Results

### GSTM1 decreased markedly in injured CFs both in vivo and in vitro

**Fig. 1:**
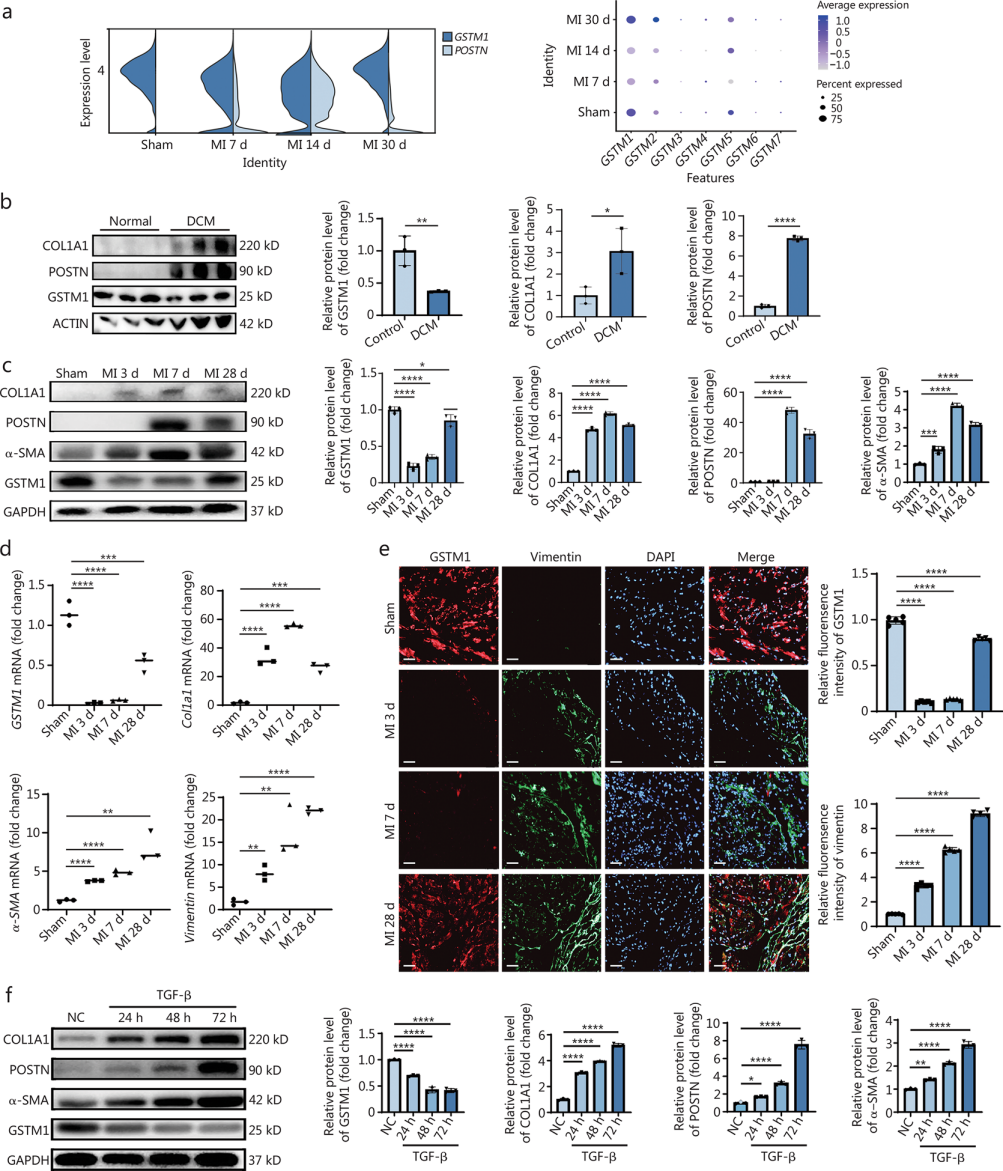
GSTM1 decreased significantly in injured cardiac fibroblasts both in vivo and in vitro. **a** The expression changes of *GSTM1* and *POSTN* in mouse fibroblasts at different time points after MI were analyzed by single-cell sequencing. **b** Western blotting analysis of the expression levels of GSTM1 and fibrosis-related indicators in normal human heart tissue and heart tissue of patients with advanced heart failure and their quantitative analysis with 3 biological repeats (*n* = 3). **c** Western blotting representative images and quantitative analysis of GSTM1 and related fibrotic markers in C57BL/6J mice in the sham group and at 3, 7, and 28 d following MI, with 3 biological repeats (*n* = 3). **d** qPCR was used to detect the changes of mRNA levels of *GSTM1* and fibrosis-related indicators in the sham group and at 3, 7, and 28 d after MI in the heart infarction area of mice with 3 biological repeats (*n* = 3). **e** Immunofluorescence staining representatives of sham group and heart infarction area at 3, 7, and 28 d after mouse MI. The red fluorescence is GSTM1, the green fluorescence is vimentin, and the blue fluorescence is DAPI (Scale bar = 50 μm). Quantitative statistics of GSTM1 and vimentin were conducted with 5 biological repeats (*n* = 5). **f** Western blotting and quantitative analysis were performed to detect GSTM1 and fibrosis index protein levels in primary mouse fibroblasts stimulated by TGF-β at different time points. Statistical differences between the two groups were determined using the Student’s *t*-test (**b**). Comparisons among three or more groups were conducted using one-way ANOVA (Fig. 1c, d, e, f). Data are presented as mean ± standard error. ^*^P < 0.05, ^**^P < 0.01, ^***^P < 0.001, ^****^P < 0.0001. NC negative control, DCM dilated cardiomyopathy, MI myocardial infarction, DAPI 4′,6-diamidino-2-phenylindole, COL1A1 collagen type I alpha 1 chain, POSTN periostin, α-SMA α-smooth muscle actin, GSTM1 glutathione S-transferase mu 1, GAPDH glyceraldehyde-3-phosphate dehydrogenase, TGF-β transforming growth factor-β

Cardiac fibrosis is characterized by the excessive accumulation of ECM following MI, resulting in adverse ventricular remodeling and detrimental heart failure [19]. ROS plays a crucial role in the pathogenesis of cardiac fibrosis [14]. Studies conducted in other organs have demonstrated that clearing intracellular ROS can effectively inhibit the progression of fibrosis. For example, in the liver, the administration of a multiple nano drug carbon quantum dot-dexamethasone (CD-Dex) was found to significantly alleviate liver damage and collagen deposition [21]. Gold-copper-based composite nanoparticles substantially enhance the survival rate of mesenchymal stem cells (MSCs) in the idiopathic pulmonary fibrosis (IPF) lung microenvironment, thereby improving the therapeutic effects of MSCs on IPF [22]. However, research on ROS clearance in post-cardiac infarction fibrosis remains limited. To gain a deeper understanding of the molecular mechanisms underlying ROS-mediated cardiac fibrosis, we analyzed the single-cell database GSE132146 to investigate the ROS system in mouse fibroblasts following MI. This analysis revealed a marked decrease in *GSTM1* expression in CFs post-MI, accompanied by an upregulation of the fibroblast activation marker *POSTN* (Fig. 1a). Additionally, the basal expression of GSTM1 in the GSTM family was the highest in CFs, and the pseudo-time series analysis showed that the changes of GSTM1 after MI were negatively correlated with the changes of fibrosis indexes (Additional file 1: Figs. S2-4).

These findings were validated using human heart samples, which were divided into the DCM group (patients exhibiting severe heart failure) and the control group. Analysis revealed that GSTM1 expression in DCM patients was significantly decreased compared with the control group, accompanied by a marked increase in the expression of fibrotic markers COL1A1 and POSTN, suggesting a potential association between cardiac fibrosis and an impaired anti-oxidant system (Fig. 1b).

To further elucidate the dynamic expression of GSTM1 in mice following MI, Western blotting, RT-qPCR, and immunofluorescence staining were employed to assess GSTM1 expression at both the mRNA and protein levels at various time points (sham, 3 d, 7 d, 28 d) after MI. The successful establishment of the MI model is verified (Additional file 1: Fig. S5). The results demonstrated a significant decrease in GSTM1 protein levels in the infarcted tissue at days 3 and 7 after MI, followed by a recovery at day 28 (Fig. 1c). Similarly, the mRNA expression of *GSTM1* reached their lowest levels on days 3 and 7 after MI (Fig. 1d). Notably, fibrotic markers increased at both mRNA and protein levels after MI, indicating CFs activation and excessive fibrosis (Fig. 1c, d). Consistently, immunofluorescence staining also confirmed a marked decrease in GSTM1 in the infarcted area on days 3 and 7 after MI, with a partial recovery on day 28 (Fig. 1e). Employing an in vitro model, adult mouse CFs were induced through TGF-β stimulation at three time points, 24, 48, and 72 h. GSTM1 expression exhibited a gradual decrease as the duration of stimulation progressed, while the fibrosis indexes (COL1A1, POSTN, and α-SMA) increased (Fig. 1f). GSTM1 expression reached its lowest level after 48 h of stimulation. These observations prompted the speculation of a negative correlation between GSTM1 and cardiac fibrosis.

### Knockdown of *GSTM1* exacerbates CF activation

**Fig. 2:**
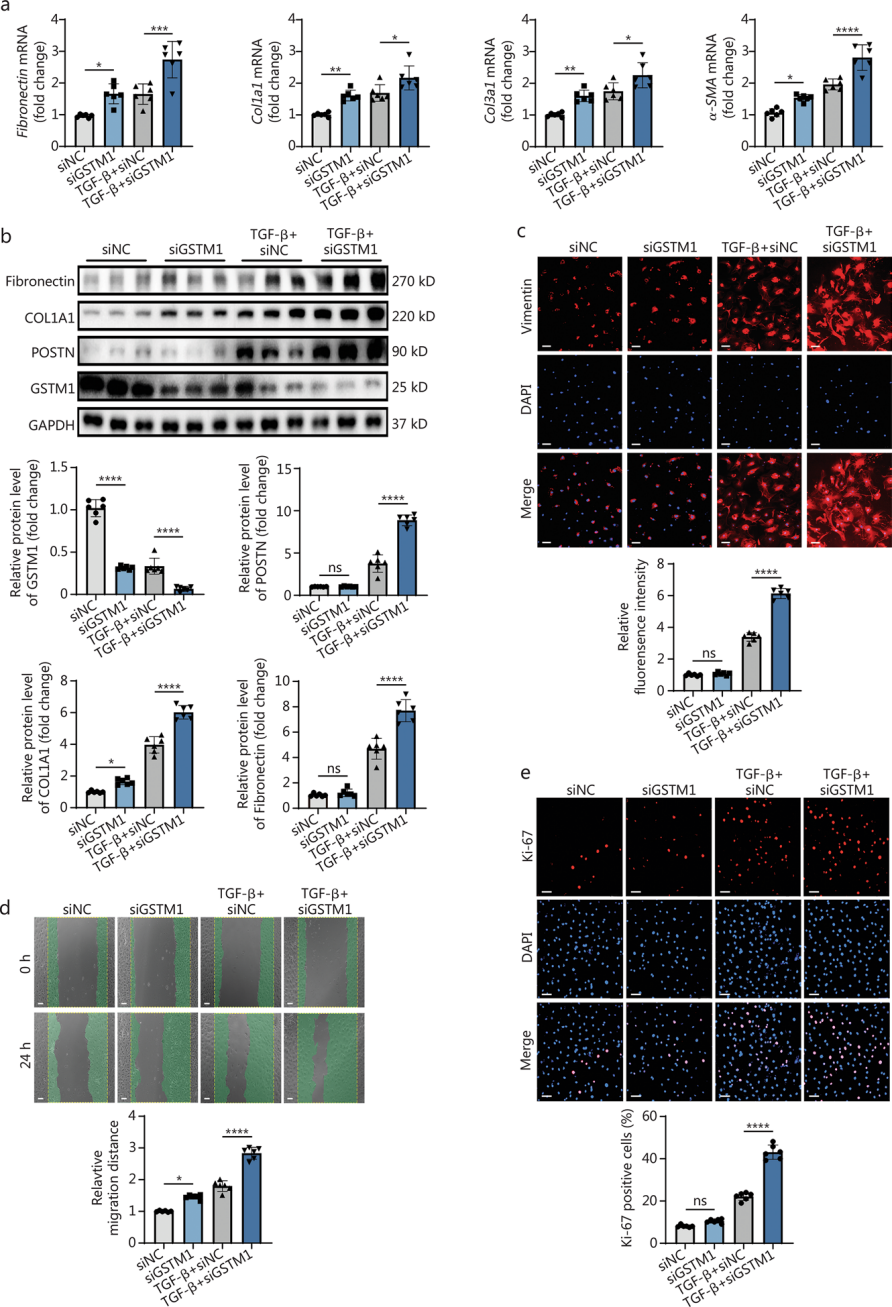
Knockdown of *GSTM1* exacerbates cardiac fibroblast activation. **a** qPCR analysis of *GSTM1* and fibrosis index mRNA levels in mouse fibroblasts stimulated by TGF-β for 48 h after siNC and siGSTM1 pretreatment, with 6 biological repeats (*n* = 6). **b** Western blotting and quantitative analysis of GSTM1 and fibrosis index protein levels in mouse fibroblasts stimulated by TGF-β for 48 h after siNC and siGSTM1 pretreatment, with 6 biological repeats (*n* = 6). **c** Immunofluorescence representation of the effect of *GSTM1* knockdown on fibroblast activation under TGF-β stimulation, vimentin was red fluorescence, and DAPI was blue fluorescence (Scale bar = 100 μm). Quantitative statistical map of relative fluorescence intensity of vimentin, the activation index of fibroblast stained by immunofluorescence, with 6 biological repeats (*n* = 6). **d** The representative map of the effect of *GSTM1* knockdown on the migration ability of cardiac fibroblasts was detected by scratch test and photographed 24 h after scratching (Scale bar = 50 μm). Quantitative statistical map of the scratch experiment: the larger the relative migration distance, the stronger the migration ability of surface cells, with 6 biological repeats (*n* = 6). **e** The immunofluorescence staining representation of *GSTM1* knockdown on cell proliferation was detected, DAPI was blue fluorescence, and Ki-67 was red fluorescence (Scale bar = 75 μm). Quantitative statistical map of Ki-67 positive cells: the more positive cells, the stronger the proliferation ability of the surface cells, with 6 biological repeats (*n* = 6). Statistical differences among the four groups were conducted using one-way ANOVA. Data are expressed as mean ± standard error. ns non-significance, ^*^P < 0.05, ^**^P < 0.01, ^***^P < 0.001, ^****^P < 0.0001. NC negative control, DAPI 4′,6-diamidino-2-phenylindole, COL1A1 collagen type I alpha 1 chain, COL3 A1 collagen type III alpha 1 chain, POSTN periostin, α-SMA α-smooth muscle actin, GSTM1 glutathione S-transferase mu 1, GAPDH glyceraldehyde-3-phosphate dehydrogenase, TGF-β transforming growth factor-β

To elucidate the role of GSTM1 in cardiac fibrosis, we employed an in vitro TGF-β stimulation model. Primary adult mouse CFs were isolated from 6- to 8-week-old C57BL/6J mice and cultured. Subsequently, small interfering RNA was utilized to knock down *GSTM1*, resulting in a 90% reduction. The CFs with *GSTM1* knockdown exhibited enhanced ECM production even without TGF-β stimulation, as indicated by upregulation of fibrotic markers at both mRNA and protein levels compared to the control group (Fig. 2a, b). CFs were then stimulated with mouse-derived TGF-β at a concentration of 10 ng/ml for 48 h. The CFs demonstrated marked upregulation of fibrotic markers as indicated by fibronectin, COL1A1, POSTN, etc., compared with the control group (Fig. 2a, b). Notably, the fibrotic markers were even more increased in CFs simultaneously stimulated with TGF-β and subjected to *GSTM1* knockdown, suggesting that *GSTM1* knockdown exacerbates CF activation and collagen production (Fig. 2a, b). Immunofluorescence staining revealed a consistent result, demonstrating a remarkable increase in vimentin expression under TGF-β stimulation with concurrent *GSTM1* knockdown in CFs (Fig. 2c).

In the context of post-infarction fibrosis, CFs proliferate, differentiate, and migrate to the scar site. To investigate the impact of GSTM1 on the function of CFs, we isolated primary adult CFs in vitro and transfected them with small interfering RNA targeting *GSTM1*. We observed that the migration capacity of CFs was enhanced upon *GSTM1* knockdown. CFs exhibited stronger migration capacity with *GSTM1* knockdown and TGF-β stimulation compared with those stimulated with TGF-β alone (Fig. 2d). Therefore, *GSTM1* knockdown promotes CF migration under both physiological and pathological conditions in vitro.

To evaluate the effect of GSTM1 on CF proliferation, we employed Ki-67 staining, a marker of proliferating cells. In the physiological state, the percentage of Ki-67^+^ fibroblasts in the *GSTM1* knockdown group did not differ from the control group (Fig. 2e). However, under TGF-β stimulation, the percentage of Ki-67^+^ fibroblasts was significantly higher in the *GSTM1* knockdown group than that in TGF-β stimulation group alone, indicating that *GSTM1* knockdown promotes proliferation in a pathological state (Fig. 2e).

Therefore, *GSTM1* knockdown promotes the activation and phenotypic transformation of CFs, as well as their proliferation and migration.

### Overexpression of GSTM1 inhibits CF activation

**Fig. 3:**
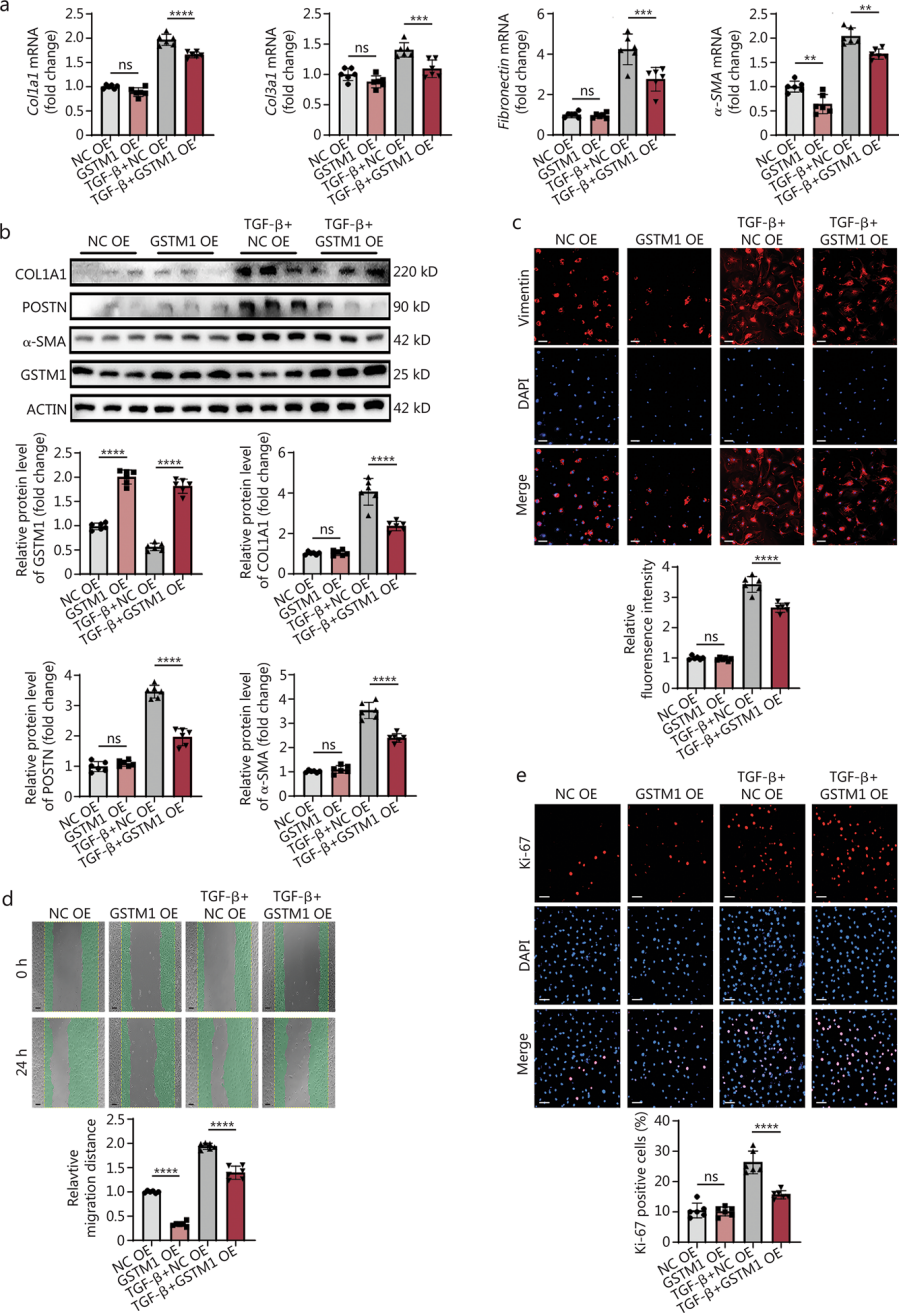
Overexpression of GSTM1 inhibits CF activation. **a** qPCR was used to detect the mRNA levels of *GSTM1* and fibrosis markers in mouse fibroblasts stimulated with TGF-β for 48 h after transfection with NC OE and GSTM1 OE plasmids, with 6 biological repeats (*n* = 6). **b** Western blotting was used to detect the protein levels of GSTM1 and fibrosis markers in mouse fibroblasts stimulated with TGF-β for 48 h after transfection with NC OE and GSTM1 OE plasmids. Quantitative analysis of protein expression levels of GSTM1, COL1A1, POSTN, and α-SMA was performed, with 3 biological repeats (*n* = 3). **c** Immunofluorescence representation of the effect of overexpression of GSTM1 on fibroblast activation under TGF-β stimulation, with red fluorescence for vimentin and blue fluorescence for DAPI. Quantitative statistical diagram of the relative fluorescence intensity of vimentin, the activation index of fibroblasts stained by immunofluorescence (Scale bar = 100 μm), with 6 biological repeats (*n* = 6). **d** The representative map of the effect of GSTM1 overexpression on the migration ability of CFs was detected by scratch test and photographed 24 h after scratching (Scale bar = 50 μm). Quantitative statistical map of the scratch experiment: the larger the relative migration distance, the stronger the migration ability of surface cells, with 3 biological repeats (*n* = 3). **e** Immunofluorescence staining representation of GSTM1 overexpression on cell proliferation, with red fluorescence for Ki-67 and blue fluorescence for DAPI. Quantitative statistical diagram of Ki-67 positive cells: the more positive cells, the stronger the proliferation ability of the surface cells (Scale bar = 75 μm), with 6 biological repeats (*n* = 6). Statistical differences among the four groups were conducted using one-way ANOVA. Data are expressed as mean ± standard error. ns non-significance, ^**^P < 0.01, ^***^P < 0.001, ^****^P < 0.0001. NC negative control, DAPI 4′,6-diamidino-2-phenylindole, COL1A1 collagen type I alpha 1 chain, COL3 A1 collagen type III alpha 1 chain, POSTN periostin, α-SMA α-smooth muscle actin, GSTM1 glutathione S-transferase mu 1, TGF-β transforming growth factor-β

Based on the findings observed under *GSTM1* knockdown, we sought to investigate whether liposome transfection-mediated overexpression of GSTM1 could mitigate fibroblast activation. GSTM1 expression was induced and elevated twofold in primary adult CFs from C57BL/6J mice aged 6–8 weeks. Compared with the TGF-β stimulation group, both mRNA and protein levels of the fibrotic markers, including COL1A1, POSTN, and α-SMA, were significantly reduced in the group with concurrent TGF-β stimulation and GSTM1 overexpression (Fig. 3a, b). Immunofluorescence staining further confirmed that the expression of vimentin was markedly lower in the GSTM1 overexpression group compared with the TGF-β stimulation group (Fig. 3c). Collectively, these findings demonstrated that overexpression of GSTM1 inhibits the activation of TGF-β-induced mouse CFs and prevents the development of fibrosis.

To further explore the effect of GSTM1 on CF behavior, GSTM1 expression was induced in primary adult mouse CFs. We found that GSTM1 overexpression suppressed the fibroblast migration under physiological conditions (Fig. 3d). Moreover, GSTM1 overexpression greatly inhibited the migration function of fibroblasts stimulated by TGF-β (Fig. 3d). To assess the effect of GSTM1 overexpression on fibroblast proliferation, we employed Ki-67 staining. The percentage of Ki-67^+^ cells did not differ between the GSTM1 overexpression group and the control group without TGF-β stimulation. However, GSTM1 overexpression markedly decreased the percentage of Ki-67^+^ cells under TGF-β stimulation, indicating that GSTM1 overexpression inhibits fibroblast proliferation in a pathological state (Fig. 3e). These findings align with the results from the *GSTM1* knockdown experiments, reinforcing the role of *GSTM1* in regulating CFs activation and phenotypic transformation.

### GSTM1 suppresses ROS production in TGF-β-induced fibroblast activation

**Fig. 4:**
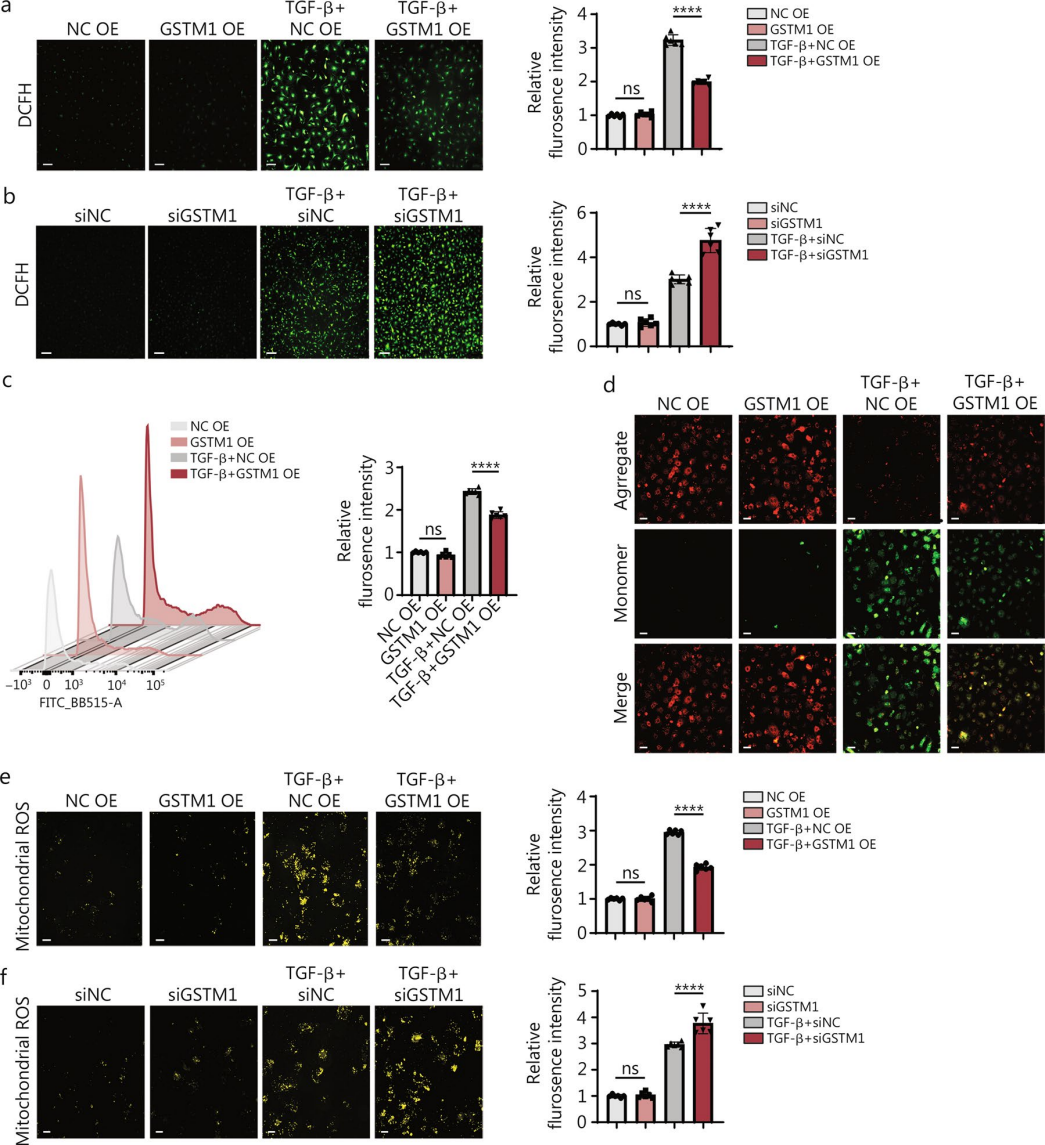
GSTM1 suppresses ROS production in TGF-β-induced fibroblast activation. **a** Intracellular ROS levels in mouse fibroblasts stimulated by TGF-β after transfection with NC OE and GSTM1 OE. The green fluorescence indicated ROS in the cytoplasm, and the higher the fluorescence intensity, the higher the ROS content in the cytoplasm (Scale bar = 50 μm). Quantitative analysis of the relative fluorescence intensity of intracellular ROS was performed, with 6 biological repeats (*n* = 6). **b** Intracellular ROS levels in mouse fibroblasts stimulated by TGF-β after transfection with si-NC and si-GSTM1. Quantitative analysis of the relative fluorescence intensity of intracellular ROS was conducted, with 6 biological repeats (*n* = 6). **c** Intracellular ROS was detected by flow cytometry. **d** After staining with JC-1 reagent, it was observed under a fluorescence microscope (red fluorescence is JC-1 polymerization, green fluorescence is JC-1 monomer) (Scale bar = 50 μm). **e** Mitochondria ROS levels in mouse fibroblasts stimulated by TGF-β after transfection with NC OE and GSTM1 OE. The yellow fluorescence represents the mitochondrial ROS content, and the stronger the fluorescence intensity, the higher the ROS production (Scale bar = 50 μm). Quantitative analysis of the relative fluorescence intensity of mitochondrial ROS was performed, with 6 biological repeats (*n* = 6). **f** Mitochondria ROS levels in mouse fibroblasts stimulated by TGF-β after transfection with si-NC and si-GSTM1 (Scale bar = 50 μm). Quantitative analysis of the relative fluorescence intensity of mitochondrial ROS was conducted, with 6 biological repeats (*n* = 6). Statistical differences among the four groups were conducted using one-way ANOVA. Data are expressed as mean ± standard error. ns non-significance, ^*^P < 0.05, ^**^P < 0.01, ^***^P < 0.001, ^****^P < 0.0001. NC negative control, DCFH 2′,7′-dichlorodihydrofluorescein diacetate, ROS Reactive oxygen species, GSTM1 glutathione S-transferase mu 1, TGF-β transforming growth factor-β

Oxidative damage resulting from MI critically impacts cardiac function, influencing various processes such as cell death, inflammatory response, and cell signal transduction. ROS, serving as a crucial second messenger in oxidative stress, can trigger a cascade of downstream molecular signaling pathways. GSTM1, a member of the GST family, plays a protective role by catalyzing the conjugation of glutathione (GSH) with electrophilic compounds, thereby protecting cells from various toxic substances. Accumulating studies have established a strong correlation between GSTM1 and oxidative stress in various contexts, such as tumor development, neurodegeneration, and so forth [23–25]. To validate this classical mechanism in CFs, we used immunofluorescence and flow cytometry to examine the impact of GSTM1 on oxidative stress. Endogenous ROS production was quantified using the DCFH probe [26]. Overexpression of GSTM1 markedly attenuated the ROS overload induced by TGF-β stimulation, whereas knockdown of *GSTM1* diminished the antioxidant capacity and increased ROS levels in fibroblasts (Fig. 4a-c).

Given the pivotal role of mitochondria as the primary sites for biological oxidation, energy metabolism, and ROS production, their dysfunction is closely associated with a myriad of diseases [27–29]. We utilized the JC-1 kit to detect changes in mitochondrial membrane potential, and the mitochondrial ROS kit to detect mitochondrial ROS production. As shown in Fig. 4d, mitochondrial membrane potential markedly decreased under TGF-β stimulation as indicated by green fluorescence from JC-1 probes. However, overexpression of GSTM1 successfully restored mitochondrial membrane potential in the activated fibroblasts. Fluorescent ROS probes also showed that TGF-β stimulation increased mitochondrial ROS production in fibroblasts, while overexpression of GSTM1 significantly reduced mitochondrial ROS levels. In contrast, the knockdown of *GSTM1* exacerbated mitochondrial ROS abnormal accumulation (Fig. 4e, f). Through these experiments, we have demonstrated that GSTM1 inhibits the production of ROS in both the cytosol and mitochondria of CFs, thereby mitigating oxidative stress.

### Lipid peroxidation and ferroptosis induce excessive activation of CFs

**Fig. 5:**
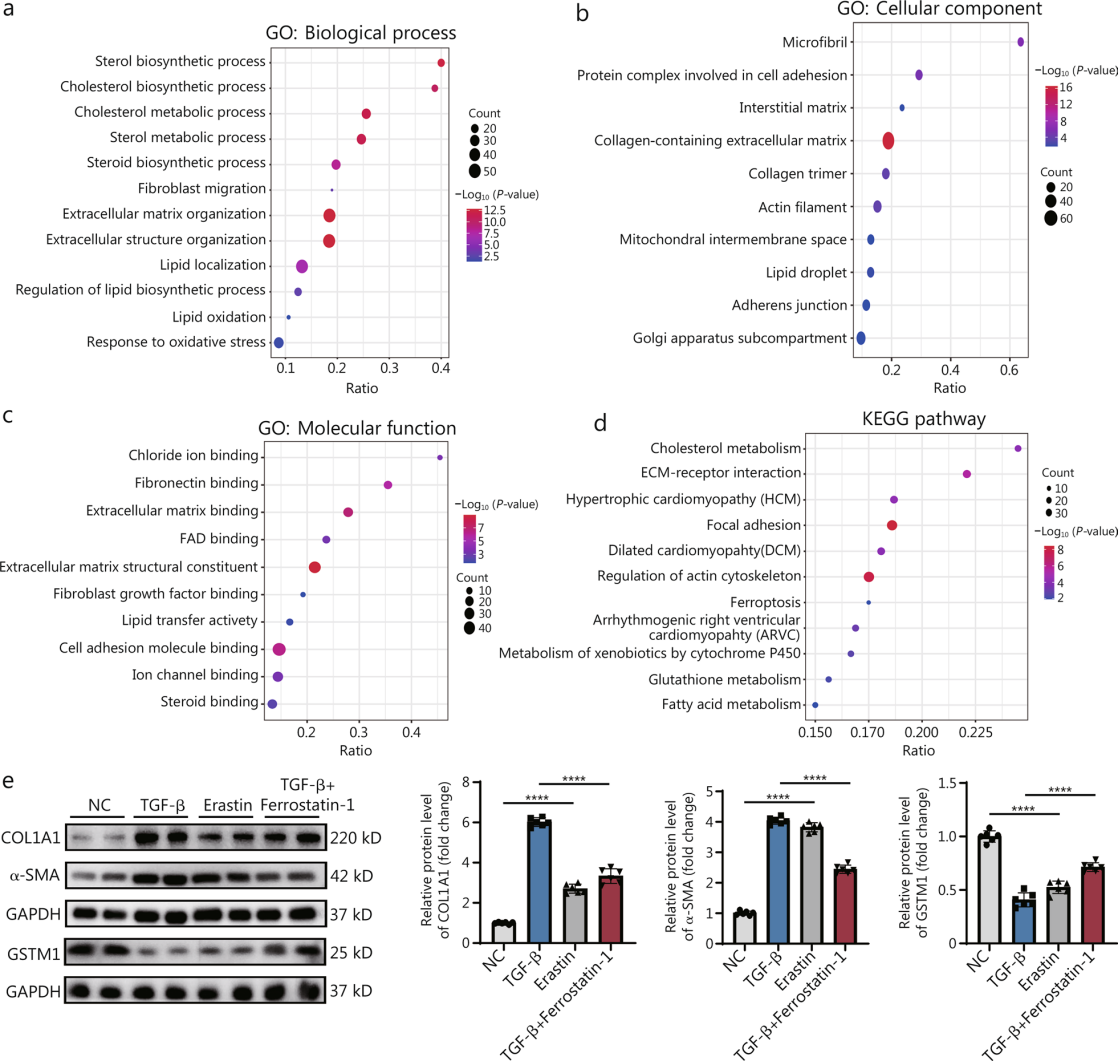
Lipid peroxidation and ferroptosis induce excessive activation of CFs. **a** GO biological process bubble map of RNA-seq sequencing analysis. **b** GO cellular component bubble map for RNA-seq analysis. **c** GO molecular function bubble map for RNA-seq analysis. **d** Bubble map of KEGG enrichment signal pathway obtained by RNA-seq analysis. **e** Fibroblasts were treated with TGF-β, ferroptosis agonist erastin, and TGF-β + ferroptosis inhibitor ferrostatin-1, and the representative graphs of fibrosis indexes were observed by Western blotting. Quantitative analysis of fibrosis-related indicators COL1A1, α-SMA, and GSTM1 was shown, with 6 biological repeats (*n* = 6). Statistical differences among the four groups were conducted using one-way ANOVA. Data are expressed as mean ± standard error. ns non-significance, ^****^P < 0.0001. NC negative control, GO Gene Ontology, KEGG Kyoto Encyclopedia of Genes and Genomes, FAD flavin adenine dinucleotide, COL1A1 collagen type I alpha 1 chain, α-SMA α-smooth muscle actin, GSTM1 glutathione S-transferase mu 1, GAPDH glyceraldehyde-3-phosphate dehydrogenase, TGF-β transforming growth factor-β

To explore the potential mechanism by which GSTM1 inhibits cardiac fibrosis, we employed RNA-seq analysis. Using Gene Ontology (GO) enrichment analysis and Kyoto Encyclopedia of Genes and Genomes (KEGG) enrichment pathway analysis, we investigated the effects of GSTM1 on CFs. The GO biological process revealed that GSTM1 primarily influenced the synthesis of ECM and cell structures associated with oxidative stress, consistent with our prior experimental findings. Additionally, our analysis indicated that GSTM1 greatly affected lipid metabolism (Fig. 5a), suggesting a potential role for GSTM1 in regulating ROS and downstream lipid peroxidation-related biological processes [30]. According to GO cellular component, GSTM1 predominantly affected cellular components associated with collagen fibers (Fig. 5b). GO molecular function showed that GSTM1 may be mainly related to lipid and collagen binding (Fig. 5c). Additionally, KEGG enrichment analysis highlighted that GSTM1 mainly regulated lipid metabolism and redox-related signaling pathways (Fig. 5d). These findings suggest a correlation among GSTM1, cardiac fibrosis, oxidative stress, and lipid metabolism. Volcano plot showed the expression of differential genes after *GSTM1* knockdown (Additional file 1: Fig. S6a). We conducted GO and KEGG enrichment analysis with up-regulated and down-regulated differential genes, respectively. Similarly, the results showed that GSTM1 was closely related to fibrosis and lipid peroxidation (Additional file 1: Fig. S6b).

Redox homeostasis and lipid metabolism are closely intertwined [31]. Oxidative stress can lead to the peroxidation of lipids, which is highly damaging to cellular membranes and may result in cell death [32]. To examine whether lipid peroxidation is elevated during fibroblast activation, oxidized lipid metabolites were profiled using oxidative lipid metabolomics. Importantly, 5-HEPE, 12-HEPE, 13-hydroxyoctadecatrienoic acid (13-HOTE), and others were markedly oxidized in CFs upon TGF-β stimulation (Additional file 1: Fig. S7). Anomalous oxidized lipids, particularly when incorporated into membrane phospholipids, can lead to ferroptosis [33]. However, the causal relationship between ferroptosis and fibroblast activation remains unclear [34]. Despite the limited evidence available, the literature presents conflicting findings regarding the potential for ferroptosis to induce fibroblast activation [35]. As shown in Fig. 5e, CFs stimulated with erastin (a ferroptosis inducer) exhibited significantly elevated levels of COL1A1 and α-SMA compared to the control group, though these levels remained slightly lower than those induced by TGF-β. When treated with ferrostatin-1 (a ferroptosis inhibitor), TGF-β-stimulated CFs showed a marked reduction in these fibrotic markers compared to TGF-β stimulation alone. Additionally, erastin stimulation significantly downregulated GSTM1 expression in fibroblasts (Fig. 5e). Intriguingly, when TGF-β stimulation was combined with ferrostatin-1, GSTM1 expression increased compared to TGF-β treatment alone. This suggests that GSTM1 may regulate fibroblast activation by modulating ferroptosis-related pathways.

### GSTM1 mitigates fibrosis by alleviating lipid peroxidation and ferroptosis

**Fig. 6:**
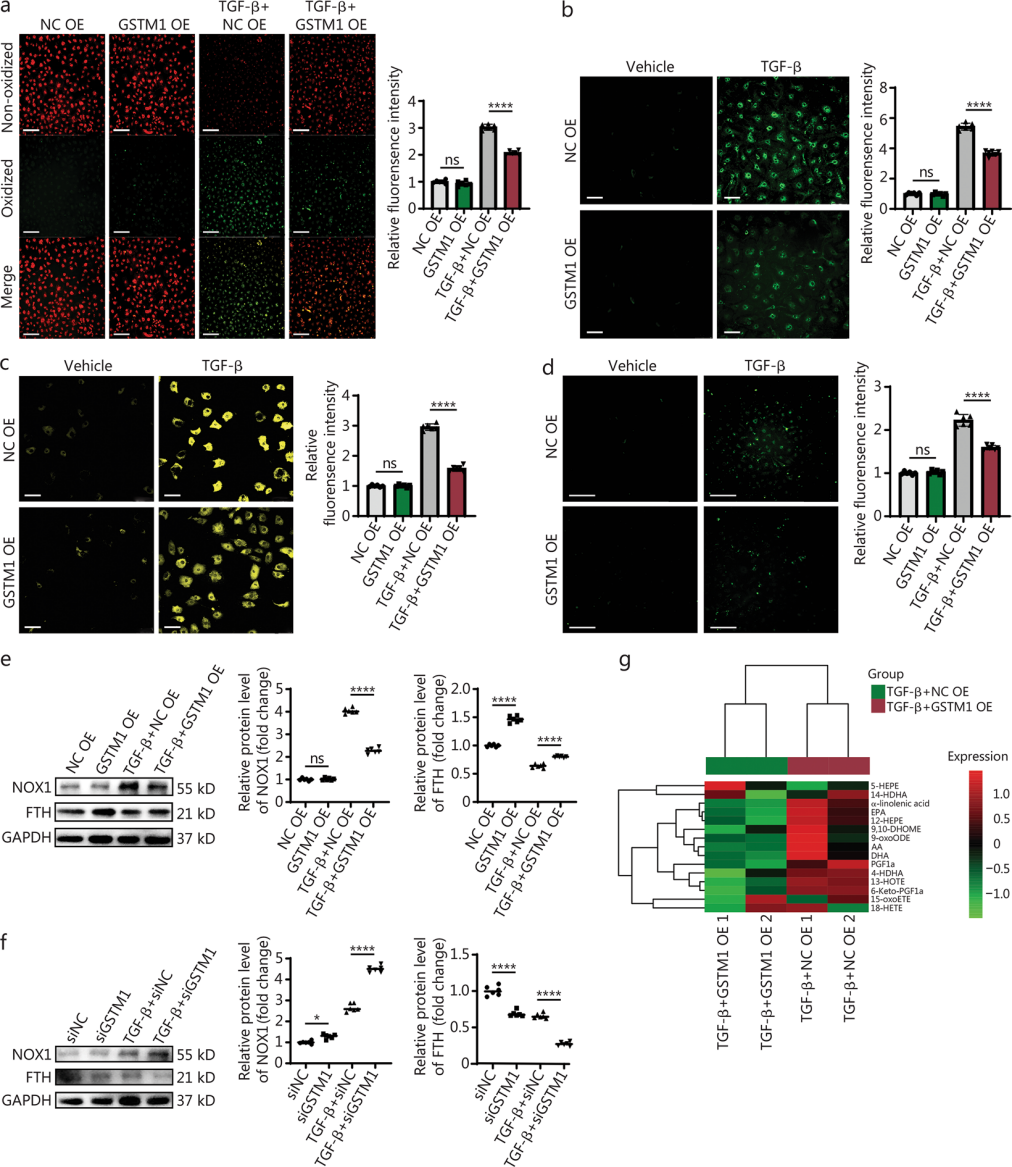
GSTM1 mitigates fibrosis by alleviating lipid peroxidation and ferroptosis.** a** Intracellular lipid peroxidation levels in mouse fibroblasts stimulated by TGF-β after transfection with NC OE and GSTM1 OE was detected by C11 probe, with red representing non-oxidized state and green representing peroxide state (Scale bar = 250 μm).** b** The fluorescence staining pattern of mitochondrial lipid peroxidation detected by MitoPeDPP probe (Scale bar = 50 μm). **c** Fluorescence staining diagram of FerroOrange probe for the detection of free ferrous ion intracellular levels (Scale bar = 50 μm). **d** Fluorescence staining diagram of Mito-FerroGreen probe for the detection of free ferrous ion levels in mitochondria (Scale bar = 250 μm). **e** Western blotting was used to detect the ferroptosis level of mouse fibroblasts stimulated by TGF-β for 48 h after pretreatment with NC OE and GSTM1 OE plasmids transfection. Quantitative statistics of NOX1 and FTH proteins were performed, with 6 biological repeats (*n* = 6). **f** Western blotting was used to detect the ferroptosis level of mouse fibroblasts stimulated by TGF-β for 48 h after pretreatment with siNC and siGSTM1. Protein quantitative statistics of NOX1 and FTH were conducted, with 6 biological repeats (*n* = 6). **g** The differential metabolite heat map of oxidative lipid metabolism between the GSTM1 overexpression and the control group. Statistical differences among the four groups were conducted using one-way ANOVA. Data are expressed as mean ± standard error. ns non-significance, ^*^P < 0.05, ^****^P < 0.0001. HDHA hydroxyacyl-CoA dehydrogenase, EPA eicosapentaenoic acid, HETE hydroxyeicosatetraenoic acid, HEPE hydroxyeicosapentaenoic acid, HOTE hydroxyoctadecatrienoic acid, ETE eicosatetraenoic acid, OXO oxoicosanoic, PG prostaglandin, ODE octadecadienoic acid, DHOME dihydroxy octadecenoic acid, DHA docosahexaenoic acid, EPA eicosapentaenoic acid, AA arachidonic acid, NC negative control, FTH ferritin heavy chain, NOX1 NADPH oxidase 1, GSTM1 glutathione S-transferase mu 1, GAPDH glyceraldehyde-3-phosphate dehydrogenase, TGF-β transforming growth factor-β

To elucidate the mechanism by which GSTM1 attenuates fibrosis through mitigating lipid peroxidation and ferroptosis, we assessed lipid peroxidation levels within the intracellular and mitochondrial compartments using the C11 probe and MitoPeDPP probe (Fig. 6a, b). The results revealed a marked elevation in both intracellular and mitochondrial lipid peroxidation upon TGF-β stimulation, which was significantly attenuated following GSTM1 overexpression. This suggests that GSTM1 effectively inhibits lipid peroxidation in fibrosis. Additionally, as free ferrous ions are critical markers of ferroptosis, we used ferro orange and ferro green probes to quantify these ions in the cytosol and mitochondria of fibroblasts (Fig. 6c, d). Overexpression of GSTM1 notably reduced the levels of free ferrous ions in both intracellular and mitochondrial compartments under TGF-β stimulation.

Western blotting additionally demonstrated that GSTM1 overexpression suppressed NOX1 expression and induced FTH expression, respectively (Fig. 6e). NOX1, a NADPH oxidase, is implicated in ferroptosis, while FTH, the heavy chain of ferritin, sequesters free ferrous ions, thereby protecting cells from ferroptosis-induced damage. Thus, GSTM1 not only alleviated oxidative stress but also reduced free ferrous ion levels, thereby inhibiting iron-dependent lipid peroxidation and the associated ferroptosis pathways. Conversely, *GSTM1* knockdown resulted in a significant upregulation of NOX1 protein and a decrease of FTH protein, consistent with the results from the overexpression experiment (Fig. 6f).

Moreover, we conducted oxidative lipid metabolome assays to investigate the impact of GSTM1 on lipid oxidation during fibroblast activation. Our results indicated that GSTM1 overexpression markedly decreased the levels of oxidized lipid metabolites, such as 12-HEPE and 13-HOTE, compared with the control group (Fig. 6g). Oxidizing lipids such as HETE have been found to reduce GSH and promote the occurrence of ferroptosis [36]. This underscores the key role of GSTM1 in regulating lipid metabolism and oxidative stress.

Therefore, these findings demonstrate that GSTM1 effectively diminishes lipid peroxidation and ferroptosis levels during fibrosis.

### GSTM1 induces GPX4 expression through the GSH/ROS/STAT3 pathway

**Fig. 7:**
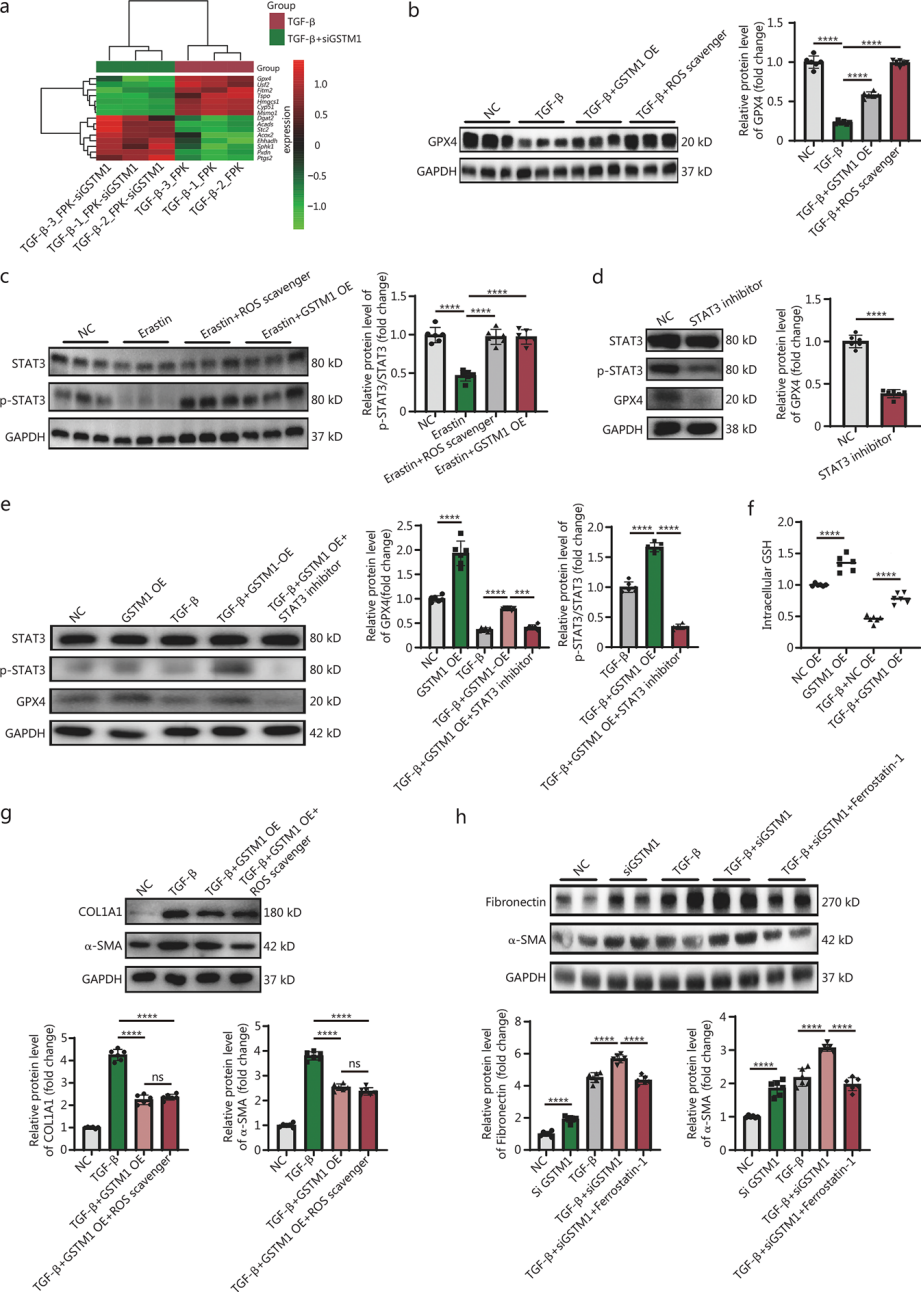
GSTM1 induces GPX4 expression through the GSH/ROS/STAT3 pathway. **a** Heatmap of differential genes associated with lipid peroxidation obtained by RNA-seq analysis. **b** Western blotting and quantitative analysis of the effects of GPX4 on TGF-β stimulation with ROS and overexpressed GSTM1, with 6 biological repeats (*n* = 6). **c** The effect of ROS on STAT3 phosphorylation was detected by Western blotting. Quantitative analysis of proteins was performed with 6 biological repeats (*n* = 6). **d** Direct effect of STAT3 dephosphorylation on GPX4. Quantitative analysis of proteins was performed with 6 biological repeats (*n* = 6). **e** Effect of GSTM1 on STAT3/GPX4 pathway. Quantitative analysis of proteins was performed with 6 biological repeats (*n* = 6). **f** The effect of overexpression of GSTM1 on GSH production in primary fibroblasts under physiological conditions and in the pathological fibrosis model was detected by the kit. **g** ROS scavenger combination with GSTM1 overexpression. Quantitative analysis of proteins was performed with 6 biological repeats (*n* = 6). **h** Western blotting analysis to determine whether GSTM1 inhibits fibrosis by interfering with lipid peroxidation. Quantitative analysis of proteins was performed with 6 biological repeats (*n* = 6). Comparisons among three or more groups were conducted using one-way ANOVA. Data are expressed as mean ± standard error. ns non-significance, ^***^P < 0.001, ^****^P < 0.0001. NC negative control, GSH glutathione, ROS reactive oxygen species, GSTM1 glutathione S-transferase mu 1, STAT3 signal transducer and activator of transcription 3, GPX4 glutathione peroxidase 4, GAPDH glyceraldehyde-3-phosphate dehydrogenase, Usf2 upstream transcription factor 2, Fitm2 fat storage inducing transmembrane protein 2, Tspo translocator protein, Hmgcs1 3-hydroxy-3-methylglutaryl-CoA synthase 1, Cyp51 cytochrome P450, family 51, Msmo1 methylsterol monooxygenase 1, Dgat2 diacylglycerol O-acyltransferase 2, Acads acyl-CoA dehydrogenase short chain, Stc2 stanniocalcin 2, Acox2 acyl-CoA oxidase 2, Ehhadh enoyl-CoA hydratase and 3-hydroxyacyl CoA dehydrogenase, Sphk1 sphingosine kinase 1, Pxdn peroxidasin, Ptgs2 prostaglandin-endoperoxide synthase 2, TGF-β transforming growth factor-β

To elucidate the molecular mechanism underlying GSTM1 regulation of lipid peroxidation and its impact on fibrosis, we conducted RNA-seq analysis. All the important genes were verified by qPCR (Fig. 7a and Additional file 1: Fig. S8). The results revealed that *Gpx4* was among the major differentially expressed genes. GPX4, a key regulator of lipid peroxidation, catalyzes the conversion of intracellular lipid peroxides to non-toxic lipid alcohols using glutathione [37]. Its significance has been well recognized across a spectrum of diseases, such as neurodegenerative disorders, malignancies, and cerebral traumas [38]. Our data demonstrated that GPX4 expression was notably diminished under TGF-β-induced fibroblast activation, which was markedly restored by GSTM1 overexpression (Fig. 7b). This suggests a potential regulatory role of GSTM1 in regulating GPX4.

STAT3 is a crucial transcription factor mediating gene expression in response to oxidative stimuli [11, 39]. Phosphorylated STAT3 can bind to the GPX4 promoter region to upregulate GPX4 transcription and expression [40]. Our findings revealed that erastin stimulation inhibited STAT3 phosphorylation in fibroblasts, whereas GSTM1 overexpression augmented the phosphorylation of STAT3 (Fig. 7c). This subsequently attenuated fibroblast activation, as indicated by decreased POSTN, COL1A1, and α-SMA expressions (Fig. 3a, b). Notably, the addition of STAT3 phosphorylation inhibitors abrogated the ability of GSTM1 overexpression to increase GPX4 expression (Fig. 7d, e).

As a glutathione transferase, GSTM1 catalyzes the synthesis of reduced GSH, a potent antioxidant capable of scavenging cellular ROS [41, 42]. Our results showed that GSTM1 overexpression enhanced GSH production and reduced ROS levels during fibroblast activation (Fig. 7f). Consistently, ROS inhibited STAT3 phosphorylation in fibroblasts, GSTM1 overexpression increased the phosphorylation of STAT3 through inhibiting ROS (Fig. 7c). There is no statistically significant difference in the degree of fibrosis in combination with ROS scavenger after GSTM1 overexpression compared with GSTM1 overexpression alone. GSTM1 causes downstream pathway changes through ROS clearance, and ultimately reduces the occurrence and development of fibrosis (Fig. 7g).

To substantiate the role of GSTM1 in fibrosis through ferroptosis, we further tested the effects of GSTM1 modulation on ferroptosis. As we demonstrated previously, *GSTM1* knockdown exacerbated fibrosis under TGF-β stimulation (Fig. 2a, b). However, the fibrosis severity in the *GSTM1* knockdown group treated with ferrostatin-1, a ferroptosis inhibitor, was attenuated compared with the *GSTM1* knockdown group alone, (Fig. 7h), indicating that GSTM1 indeed inhibits fibrosis through lipid peroxidation and ferroptosis.

Therefore, our data suggest that GSTM1 diminishes ROS levels by augmenting GSH production, enhancing the phosphorylation of STAT3, and promoting GPX4 expression. This cascade ultimately inhibits lipid peroxidation and ferroptosis, alleviating fibrosis.

### AAV-mediated GSTM1 overexpression protects infarcted hearts by alleviating fibrosis

To elucidate the protective role of GSTM1 in MI, we engineered an AAV9 to overexpress mouse GSTM1. AAV9-GSTM1 was injected through the tail vein. Overexpression efficiency was verified in Additional file 1: Fig. S9. Echocardiographic analysis revealed no significant change in the basic cardiac function of the overexpression group. There were no significant differences in baseline cardiac structure and function between the GSTM1 overexpression group and control group, with no difference in left ventricular ejection fraction (EF), fraction shortening (FS), left ventricular internal dimension at end-diastole (LVIDd) and left ventricular internal dimension at end-systole (LVIDs) (Additional file 1: Fig. S10a). There is also no significant effect on the electrocardiogram (ECG) changes in the acute phase of MI (Additional file 1: Fig. S10b). Additionally, histological examination using HE staining and general cardiogram also indicated consistent heart size (Additional file 1: Fig. S10c).

**Fig. 8:**
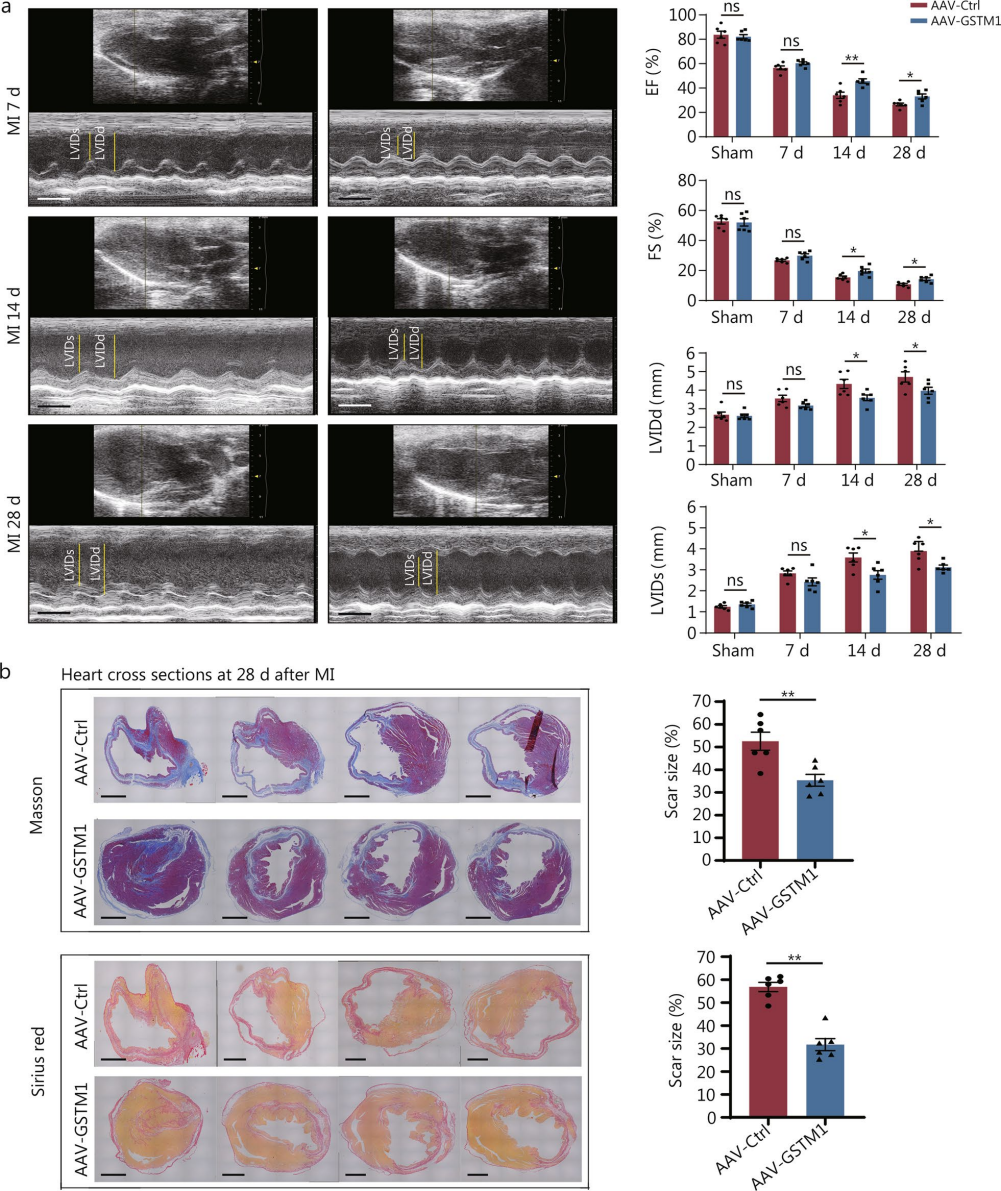
AAV-mediated GSTM1 overexpression protects infarcted hearts by alleviating fibrosis.** a** Echocardiography at 7, 14, and 28 d after MI of mice with over-expression of GSTM1-AAV9 virus injected in the tail vein compared with the negative injection control. Timestamp = 100 ms. Echogram of mice ventricular ejection fraction (EF), fraction shorting (FS), left ventricular internal dimension at end-diastole (LVIDd), and left ventricular internal dimension at end-systole (LVIDs), with 6 biological repeats (*n* = 6). **b** Masson and Sirius red staining maps and statistical maps of infarct size were performed on the heart tissues of mice 28 d after myocardial infarction (Scale bar = 1 mm), with 6 biological repeats (*n* = 6). Statistical differences between the two groups were determined using the Student’s *t*-test. Data are expressed as mean ± standard error. ns non-significance, ^*^P < 0.05, ^**^P < 0.01. AAV adeno-associated virus, MI myocardial infarction, Ctrl control, GSTM1 glutathione S-transferase mu 1

Serial echocardiography was carried out to monitor cardiac function at 7, 14, and 28 d post-MI. At 28 d, mice overexpressing GSTM1 exhibited greatly improved cardiac function compared to that of the control group, with elevated EF and FS, and reduced LVIDd and LVIDs (Fig. 8a). This suggests that GSTM1 overexpression ameliorates cardiac dysfunction after MI in mice. The survival rate of the experimental group was significantly improved compared with the control group after MI. (Additional file 1: Fig. S11). Sirius red and Masson staining revealed significantly reduced infarct size and attenuated fibrosis (Fig. 8b). Taken together, these data provide insights into the therapeutic potential of GSTM1 in cardiac fibrosis.

## Discussion

Adverse ventricular remodeling following MI leads to reduced ventricular compliance and the progression of heart failure, primarily driven by fibrosis [43]. Our study provides new evidence of GSTM1’s role in mitigating cardiac fibrosis by countering intracellular oxidative stress and reducing lipid peroxidation. Overexpressing GSTM1 alleviates post-MI fibrosis, offering a novel perspective for preventing heart failure.

GSTM1, a key member of the GST family, functions in detoxification and antioxidant stress through glutathione conjugation [44, 45]. Clinical studies have suggested a potential association between the GSTM1 null polymorphism and increased susceptibility to cardiovascular diseases, including hypertension, atherosclerosis, and heart failure [46, 47]. Our findings indicate a significant downregulation of GSTM1 in mouse CFs after MI and in heart tissues from patients with DCM in advanced heart failure. Previous reports have associated GSTM1 deficiency with various fibrotic diseases, such as oral mucosal fibrosis, cystic fibrosis, and cirrhosis, suggesting a broader role of GSTM1 in regulating fibrosis [48–50]. Our data demonstrates that GSTM1 inhibits collagen synthesis, fibroblast proliferation, and migration and prevents fibroblast-to-myofibroblast transformation.

Ferroptosis, a newly characterized form of programmed cell death associated with intracellular iron overload and lipid peroxidation [51, 52], has been implicated in cardiovascular diseases such as hypertension, atherosclerosis, and cardiomyopathy [53]. In the liver, iron overload promotes liver cell ferroptosis, subsequently resulting in liver injury and fibrosis [54]. Elevated iron levels have been observed in idiopathic pulmonary fibrosis, correlating with increased fibroblast proliferation and ECM synthesis [55]. TGF-β has been shown to upregulate transferrin receptor expression, leading to iron accumulation and activation of human lung fibroblast cell lines [56]. A previous study indicates that SIRT7 reduces renal fibrosis by mitigating renal ferroptosis in hypertensive patients [57]. Conversely, ferroptosis may also lead to decreased fibrosis, celastrol exhibits anti-fibrotic effects via inducing ferroptosis in activated hepatic stellate cells [58]. These studies suggest a complicated role of ferroptosis in fibrotic diseases.

In cardiac studies, ferroptosis has been primarily investigated in cardiomyocyte death with iron accumulation following MI [40, 41]. GSTM1 may also improve cardiac function by inhibiting ferroptosis of cardiomyocytes after MI (Additional file 1: Fig. S12). However, the relationship between ferroptosis and cardiac fibrosis remains unexplored. Our data reveals a crucial role of GSTM1 in collagen synthesis and fibroblast phenotypic transformation through lipid metabolism and oxidative stress. We identified the differentially expressed genes related to lipid peroxidation downstream of GSTM1, including upregulation of *Dgat2*, *Acads, Stc2, Acox2, Ehhadh, Sphk1, Pxdn, Ptgs2*, and downregulation of *Usf2, Fitm2, Tspo, Hmgcs1, Msmo1*. GPX4, a key inhibitor of lipid peroxidation, shows markedly downregulation in the setting of *GSTM1* knockdown. Among the 8 isoenzymes (GPX1–8) in the GPX family, GPX4 exhibits an extraordinary inhibitory role in lipid peroxidation [38]. It reduces lipid peroxides to lipid alcohols via GSH [59]. This led us to further explore the role of the GSTM1-GPX4 axis in regulating cardiac fibrosis.

Additionally, our oxidative lipid metabolome assay revealed that GSTM1 significantly reduces levels of oxidized lipids such as hydroxyeicosapentaenoic acid (HEPE), docosahexaenoic acid (DHA), oxooctadecadienoic acid (oxoODE), improving lipid metabolism and alleviating lipid peroxidation. Oxidized lipids, produced by the autooxidation of polyunsaturated fatty acids [arachidonic acid (AA), alpha-linolenic acid, DHA, Eicosapentaenoic acid (EPA), etc.] or by enzyme action, have been linked to cardiovascular diseases [60]. For example, cyclooxygenase (COX) converts arachidonic acid to 9-alpha-dimorphecolic acid (9-HODE), while lipoxygenase (LOX) metabolizes it to form medium-chain hydroxyeicosatetraenoic acid (HETE). CytochromeP (CYP) metabolizes AA to produce ω-terminal (16-, 17-, 18-, 19-, and 20-) HETE or epoxyeicosatrienoic acid (EETs). Oxidized lipids have a wide range of biological functions in the cardiovascular system [61]. At present, a considerable number of studies have shown that many oxidized lipid metabolites are believed to be related to cardiovascular diseases, fibrosis, and ferroptosis [62]. Our oxidized lipid metabolome has detected several differential metabolites, all of which may play a potential role in ferroptosis during fibrosis. According to relevant studies, HETE/HEPE is produced by the oxidation of AA by lipoxygenase. In the mouse model of cerebral hemorrhage, 20-HETE can inhibit the expression of GPX4, thus aggravating oxidative stress, promoting the occurrence and development of iron death, and aggravating acute brain injury [63]. In the sepsis model, 20-HETE inhibitors can reduce the process of ferroptosis induced by STING [64]. In nonalcoholic steatohepatitis, mitotic protein 2 (Mfn2) promotes ferroptosis by interacting with inositol demand enzyme (IRE1α) to promote the production of 5-HETE and ferroptosis and hepatitis is significantly alleviated by reducing the content of 5-HETE [65]. In renal cell carcinoma, 5-, 12-, and 15-HETE can be induced by lycorine to increase their expression, contributing to the occurrence of ferroptosis [36]. However, the role of a substantial portion of oxidized lipids in ferroptosis remains unclear, and these may be potential targets. Accordingly, we will conduct more in-depth studies on these differential metabolites in the future. It is important to note that there have been studies that have reported the relationship between sex and ferroptosis before, and estrogen may interfere with the progression of ferroptosis in some way [66, 67]. We also conducted experiments using female mice and found that overexpression of GSTM1 similarly improved cardiac function and reduced fibrosis in female mice after MI (Additional file 1: Figs. S13, S14). However, the long-term effects and whether estrogen influences the intervention of GSTM1 on the ferroptosis pathway remain unclear. This necessitates further experimental exploration in the future.

The recombinant AAV vector used in this study has become an important tool in gene therapy due to its low immunogenicity and long-term gene expression characteristics. However, off-target effects may occur through 2 main pathways. (1) Tissue/cell-level off-targeting: the vector may enter non-target tissues through systemic circulation or local diffusion, potentially leading to transgene expression in unintended tissues; (2) Genome-level off-targeting: the recombinant AAV vector DNA may randomly integrate into the host genome, potentially disrupting critical genes or regulatory regions, and posing a risk of insertional mutations [68, 69]. Based on the literature and the experimental design of this study, the AAV9 serotype used here has a demonstrated advantage in cardiac-specific transduction. However, AAV9 also exhibits a high affinity for skeletal muscle, the liver, and the central nervous system, which may lead to transgene expression in these non-target tissues. Additionally, high-dose systemic administration may increase the risk of off-target effects. Although this study employed tail vein injection and minimized the dose (1 × 10^12^ vg/ml, 100 μl per mouse) while achieving successful overexpression in the target tissue, off-target risks remain. Furthermore, the study lacks long-term safety assessments: short-term observations in animal models (less than 2 months in this study) may not be sufficient to capture delayed off-target effects, necessitating long-term follow-up studies in the future. To enhance the safety of future clinical applications, we propose the following improvements. (1) Capsid engineering: develop novel capsids with enhanced tissue specificity through directed evolution or computer-aided design [70]; (2) Regulatory element optimization: incorporate microRNA-responsive elements to suppress transgene expression in non-target tissues; (3) Precision delivery techniques: utilize image-guided or organ-specific delivery devices (ultrasound-targeted microbubble destruction) to increase local vector concentration [71, 72].

## Conclusions

**Fig. 9:**
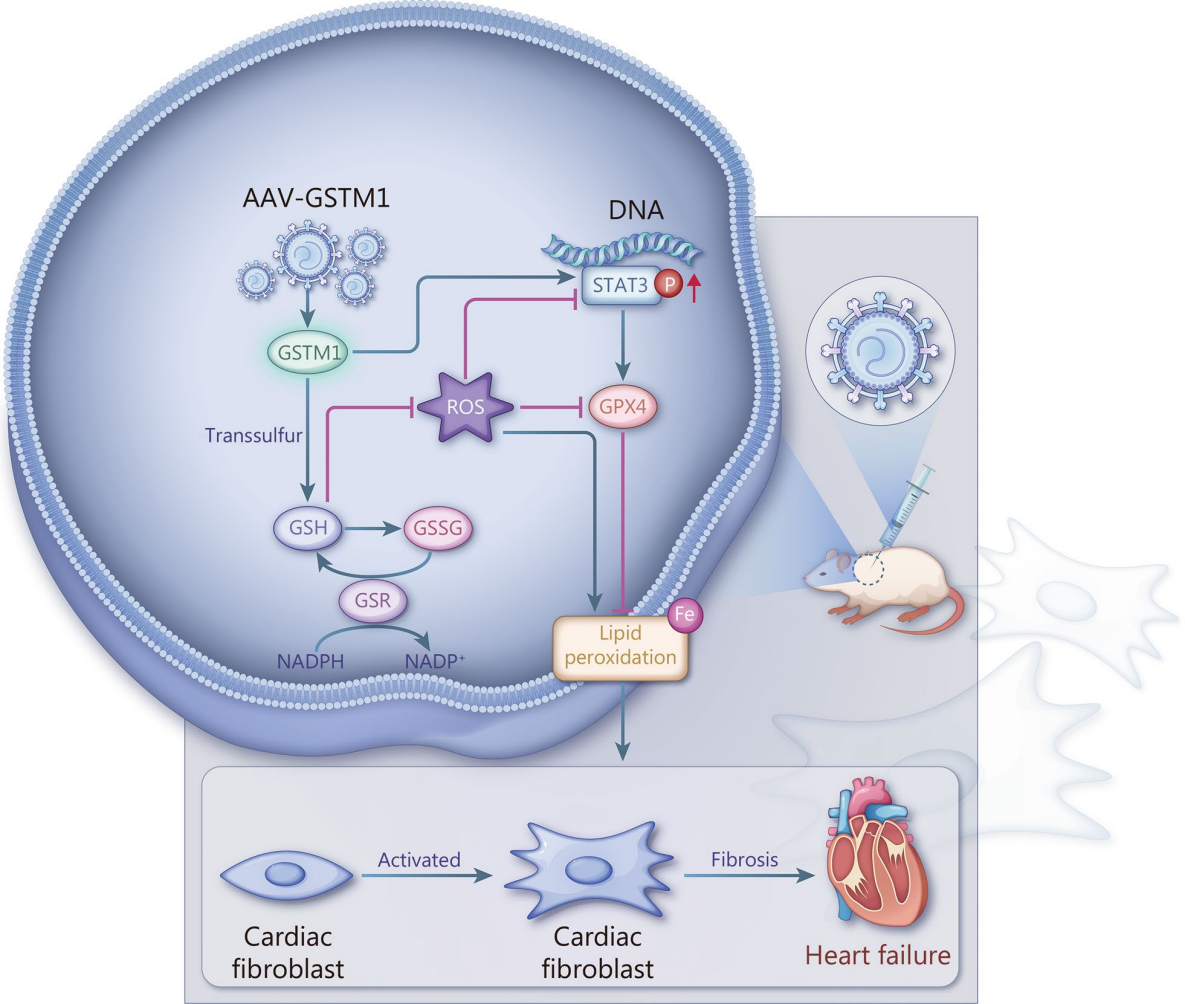
By intervening in the GSH/ROS/STAT3/GPX4 pathway in cardiac fibroblasts, GSTM1 inhibits the production of lipid peroxidation, thus inhibiting the activation and phenotype transformation of fibroblasts, improving the cardiac function after myocardial infarction and alleviating the occurrence and development of fibrosis. GSTM1 glutathione S-transferase mu 1, GSH glutathione, ROS reactive oxygen species, GPX4 glutathione peroxidase 4, GSSG oxidized glutathione, GSR glutathione reductase, STAT3 signal transducer and activator of transcription 3

In summary, our study elucidates the critical role of GSTM1 in the pathogenesis of cardiac fibrosis following MI. GSTM1 overexpression significantly improves cardiac function and alleviates fibrosis. GSTM1 suppresses fibroblast activation, phenotypic transformation, proliferation, and migration by reducing lipid peroxidation in CFs. GSTM1 exerts its role by alleviating lipid peroxidation via the GSH/ROS/STAT3/GPX4 pathway (Fig. 9). However, there remain unresolved questions to fully delineate the regulatory network of GSTM1. Future research may pave the way for the application of GSTM1-based therapy to prevent adverse ventricular remodeling and heart failure.

## Supplementary Information

**Additional file 1.** Methods. **Table S1** Primer sequence of the target gene. **Table S2** Antibody information used in the experiment. **Fig. S1** Layered heart slices. **Fig. S2** Cardiac fibroblasts were divided into different subgroups and the changes of each subgroup after myocardial infarction were analyzed. **Fig. S3** Single-cell analysis. **Fig. S4** Single-cell analysis. **Fig. S5** Identification of myocardial infarction model. **Fig. S6** KEGG and GO analysis of differentially expressed genes. **Fig. S7** Metabolite heat map. **Fig. S8** PCR validation of differentially expressed genes associated with lipid peroxidation identified by RNA-seq. **Fig. S9** Validation of AAV-mediated GSTM1 overexpression efficiency. **Fig. S10** Cardiac function, electrocardiogram, and cardiac morphology in GSTM1-AAV9 and GSTM1-Ctrl mice at baseline. **Fig. S11** Survival curve of mice after myocardial infarction. **Fig. S12** Effects of GSTM1 on oxidative stress and ferroptosis in cardiomyocytes. **Fig. S13** AAV-mediated GSTM1 overexpression protects infarcted hearts in female mice. **Fig. S14** AAV-mediated GSTM1 overexpression alleviated cardiac fibrosis after MI in female mice.

## Data Availability

All data are available upon request from the corresponding author (Hong Ma).

## Abbreviations

AA: Arachidonic acid
AAV: Adeno-associated virus
CD-Dex: Carbon quantum dot-dexamethasone
CFs: Cardiac fibroblasts
COX: Cyclooxygenase
CYP: CytochromeP
DCM: Dilated cardiomyopathy
DHA: Docosahexaenoic acid
ECM: Extracellular matrix
EET: Epoxyeicosatrienoic acid
EPA: Eicosapentaenoic acid
GSH: Glutathione
GSTs: Glutathione S-transferases
GO: Gene Ontology
HEPE: Hydroxyeicosapentaenoic acid
HODE: Alpha-dimorphecolic acid
HOTE: Hydroxyoctadecatrienoic acid
IPF: Idiopathic pulmonary fibrosis
KEGG: Kyoto Encyclopedia of Genes and Genomes
MI: Myocardial infarction
MSCs: Mesenchymal stem cells
oxoODE: Oxooctadecadienoic acid
ROS: Reactive oxygen species
RT-qPCR: Real-time quantitative polymerase chain reaction
TGF-β: Transforming growth factor-β
