# Supplementary material for: GSTM1 suppresses cardiac fibrosis post-myocardial infarction through inhibiting lipid peroxidation and ferroptosis
Source: Mil Med Res. 2025 May 31;12:26. doi: 10.1186/s40779-025-00610-6 (PMC12125851; doi:10.1186/s40779-025-00610-6)
Supplement: Supplementary file 1 — Additional file 1. Methods. Table S1 Primer sequence of the target gene. Table S2 Antibody information used in the experiment. Fig. S1 Layered heart slices. Fig. S2 Cardiac fibroblasts were divided into different subgroups and the changes of each subgroup after myocardial infarction were analyzed. Fig. S3 Single-cell analysis. Fig. S4 Single-cell analysis. Fig. S5 Identification of myocardial infarction model. Fig. S6 KEGG and GO analysis of differentially expressed genes. Fig. S7 Metabolite heat map. Fig. S8 PCR validation of differentially expressed genes associated with lipid peroxidation identified by RNA-seq. Fig. S9 Validation of AAV-mediated GSTM1 overexpression efficiency. Fig. S10 Cardiac function, electrocardiogram, and cardiac morphology in GSTM1-AAV9 and GSTM1-Ctrl mice at baseline. Fig. S11 Survival curve of mice after myocardial infarction. Fig. S12 Effects of GSTM1 on oxidative stress and ferroptosis in cardiomyocytes. Fig. S13 AAV-mediated GSTM1 overexpression protects infarcted hearts in female mice. Fig. S14 AAV-mediated GSTM1 overexpression alleviated cardiac fibrosis after MI in female mice. [file 40779_2025_610_MOESM1_ESM.pdf]

Methods

Reactive oxygen species (ROS), lipid peroxidation, and ferroptosis assessment

The 2',7'-dichlorodihydrofluorescein diacetate (DCFH-DA) probe (Beyotime, China) was utilized to evaluate the production of cytoplasmic ROS within cardiac fibroblasts (CFs). Concurrently, the mitochondrial ROS assay kit (Cayman, United States) was employed to detect the generation of ROS within the mitochondria of CFs. CFs were cultured and transfected according to established protocols and subsequently stimulated with TGF-β. DCFH-DA was diluted in serum-free medium at a ratio of 1:1000 and added to the cells following the removal of the culture medium. The cells were incubated at 37 °C for 20 min, after which they were subjected to 3 washes using a serum-free medium. The ROS levels were quantified by applying flow cytometry or immunofluorescence techniques.

To assess lipid peroxidation levels within CFs, BDP 581/591 C11 (Tongren Chemical, Japan) and MitoPeDPP were utilized. CFs were cultured and transfected according to previously established protocols, followed by stimulation with transforming growth factor-β (TGF-β). The C11 or MitoPeDPP probe was introduced to the cells for 15 − 30 min at 37 °C, adhering to the manufacturer's guidelines. After 2 washes with Hanks' HEPES buffer, the cells were subjected to imaging under a fluorescence microscope.

Ferro orange and mito-FerroGreen were utilized to assess the concentrations of ferrous ions within the cytosol and mitochondria of CFs, respectively. Following an incubation period of 15 − 30 min at 37 °C and 2 subsequent washes with Hanks' HEPES buffer, the cells were examined under a fluorescence microscope.

Whole genome RNA sequencing (RNA-seq) and analysis

From each sample, 1.5 μg of total RNA was extracted. The NEBNext® Ultra™ RNA Library Prep Kit

(NEB, United States) was utilized to construct sequencing libraries in accordance with the manufacturer's instructions, incorporating unique barcodes into each sample library sequence. Specifically, mRNA was isolated from the total RNA using poly-T magnetic beads. Subsequently, the mRNA was fragmented at an elevated temperature using NEBNext first strand synthesis reaction buffer (5×) and divalent cations. First-strand cDNA synthesis was initiated by adding hexamer primers and M-MuLV Reverse Transcriptase (RNase H^-). Subsequently, second-strand cDNA synthesis was carried out using DNA Polymerase I and RNase H^-. Any remaining overhangs were converted into blunt ends through exonuclease/polymerase treatment. The 3' ends of the DNA fragments were adenylated, followed by hybridization with NEBNext adaptors possessing a hairpin loop structure. To select cDNA fragments of appropriate lengths, the cDNA fragments were purified using the AMPure XP system (Beckman Coulter, United States). PCR amplification was performed by incubating 3 μl of USER enzyme (NEB, United States) and ligated cDNA with adaptors at 37 °C for 15 min, followed by 95 °C for 5 min. Phusion High-Fidelity DNA Polymerase (NEB, United States), universal PCR primers, and Index (X) primers were added during the PCR reaction. Finally, the products were purified using the AMPure XP system, and library quality was assessed using the Agilent Bioanalyzer 5400 system. Sequencing was performed on an Illumina NovaSeq 6000 platform, generating paired-end reads of 150 base pairs. The number of sequence fragments aligned to each gene was calculated using HTSeq (version 0.13.5). FPKM represents the number of reads fragments aligned to a gene per million reads fragments per kilobase of gene length. Differential expression analysis of the 2 groups of samples was conducted using the DESeq2 R package (version 1.26.0). We conducted differential expression analysis on the protein-coding genes and selected a subset of differentially expressed genes based on the criteria of $|\log_2 \text{fold change}| > 1$, P-value < 0.05, and an FPKM ≥ 1 in at least one sample (thereby excluding genes with the lowest expression levels). Building upon these differentially expressed genes,

we performed Gene Ontology (GO) enrichment analysis and Kyoto Encyclopedia of Genes and Genomes (KEGG) pathway analysis using the clusterProfiler package. We chose to display terms with a *P*-value < 0.05, a high GeneRatio ranking, and relevance to fibrosis, cardiac, and oxidative stress for presentation.

Lipid oxidation metabolomics

The samples were thawed on ice, and 350 µl of methyl tert-butyl ether along with 10 magnetic beads were added to each tube. The mixture was homogenized for 3 min (BB24, Next Advance., Inc., NY, United States) and subjected to oscillation extraction for 15 min. Subsequently, the samples were centrifuged at 18,000 × *g* for 10 min at 4 °C (Microfuge 20R, Beckman Coulter, Inc., Indianapolis, IN, United States), and 300 µl of the supernatant was transferred to a new centrifuge tube. The supernatant was then evaporated under nitrogen gas and reconstituted in 3 ml of acetonitrile. Following the activation and equilibration of Poly-Sery MAX columns, the reconstituted samples were loaded, washed, eluted, and collected. The eluate was subsequently evaporated and reconstituted in 60 µl of methanol for subsequent analysis using the Acquity-I Xevo TQ-S liquid chromatography-mass spectrometry system (Waters Corp., Milford, MA, United States) for targeted detection of specific metabolites.

Table S1 Primer sequence of the target gene

**Gene**	**Sequence (5’ – 3’)**
β-actin	GTGACGTTGACATCCGTAAAGA
	GCCGGACTCATCGTACTCC
GSTM1	ATACTGGGATACTGGAACGTCC
	AGTCAGGGTTGTAACAGAGCAT
Col1a1	GCTCCTCTTAGGGGCCACT
	CCACGTCTCACCATTGGGG
Col3a1	ACGTAGATGAATTGGGATGCAG
	GGGTTGGGGCAGTCTAGTG
Fibronectin	GGCCACCATTACTGGTCTGG
	GGAAGGGTAACCAGTTGGGG
POSTN	CCTGCCCTTATATGCTCTGCT
	AAACATGGTCAATAGGCATCACT
α-SMA	GTCCCAGACATCAGGGAGTAA
	TCGGATACTTCAGCGTCAGGA

COL1A1 collagen type I alpha 1 chain, *POSTN* periostin, *α-SMA* α-smooth muscle actin, *COL3A1* collagen type III alpha 1 chain, *GSTM1* glutathione S-transferase mu 1

Table S2 Antibody information used in the experiment

Target antigen	Vendor	Catalog	Concentration	Working
COL1A1	Cell Signaling Technology	72026	1:1000	Western blotting
POSTN	Cell Signaling Technology	20302	1:1000	Western blotting
GSTM1	Abcam	ab108524	1:1000	Western blotting
β-actin	Cell Signaling Technology	12262	1:3000	Western blotting
GAPDH	Aksomics	KC-5G5	1:3000	Western blotting
α-SMA	Invitrogen	PA5-18292	1:1000	Western blotting
Fibronectin	Abcam	ab2413	1:1000	Western blotting
Ferritin heavy chain	Abcam	ab183781	1:1000	Western blotting
NOX1	Abcam	ab131088	1:1000	Western blotting
GPX4	Abcam	ab125066	1:1000	Western blotting
STAT3	Cell Signaling Technology	12640	1:1000	Western blotting
p-STAT3	Cell Signaling Technology	9145	1:1000	Western blotting
Vimentin	Abcam	ab8978	1:200	Immunofluorescence
Ki-67	Abcam	ab15580	1:200	Immunofluorescence

COL1A1 collagen type I alpha 1 chain, *POSTN* periostin, *α-SMA* α-smooth muscle actin, *GSTM1* glutathione S-transferase mu 1, *STAT3* signal transducer and activator of transcription 3, *p-STAT3* phosphorylated-STAT3, *NOX1* NADPH oxidase 1, *GPX4* glutathione peroxidase 4, *GAPDH* glyceraldehyde-3-phosphate dehydrogenase

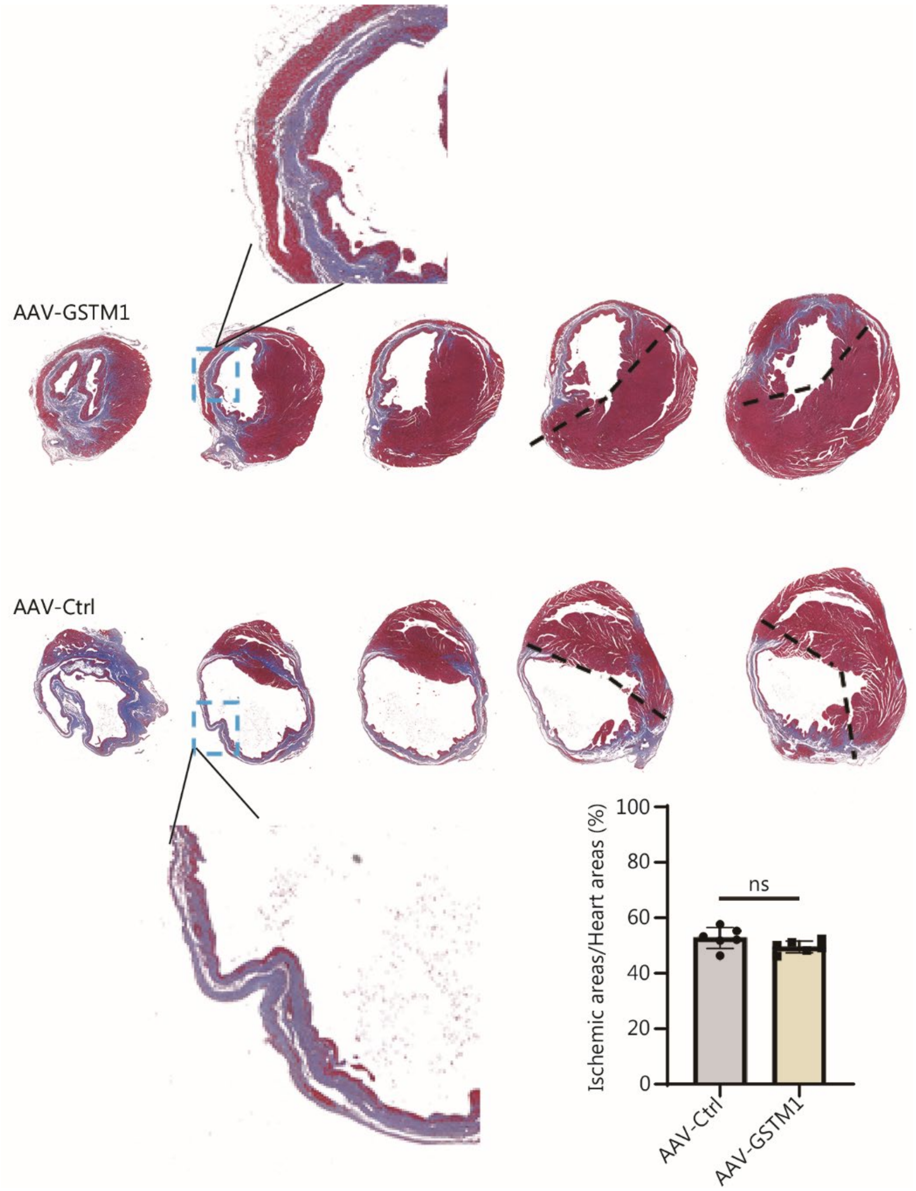

Fig. S1 Layered heart slices. The arrangement of the stratified sections from the apex to the bottom of the heart and the proportion of ischemic area in the total heart after myocardial infarction (MI) in mice. AAV adeno-associated virus, Ctrl control, GSTM1 glutathione S-transferase mu 1, ns non-significance

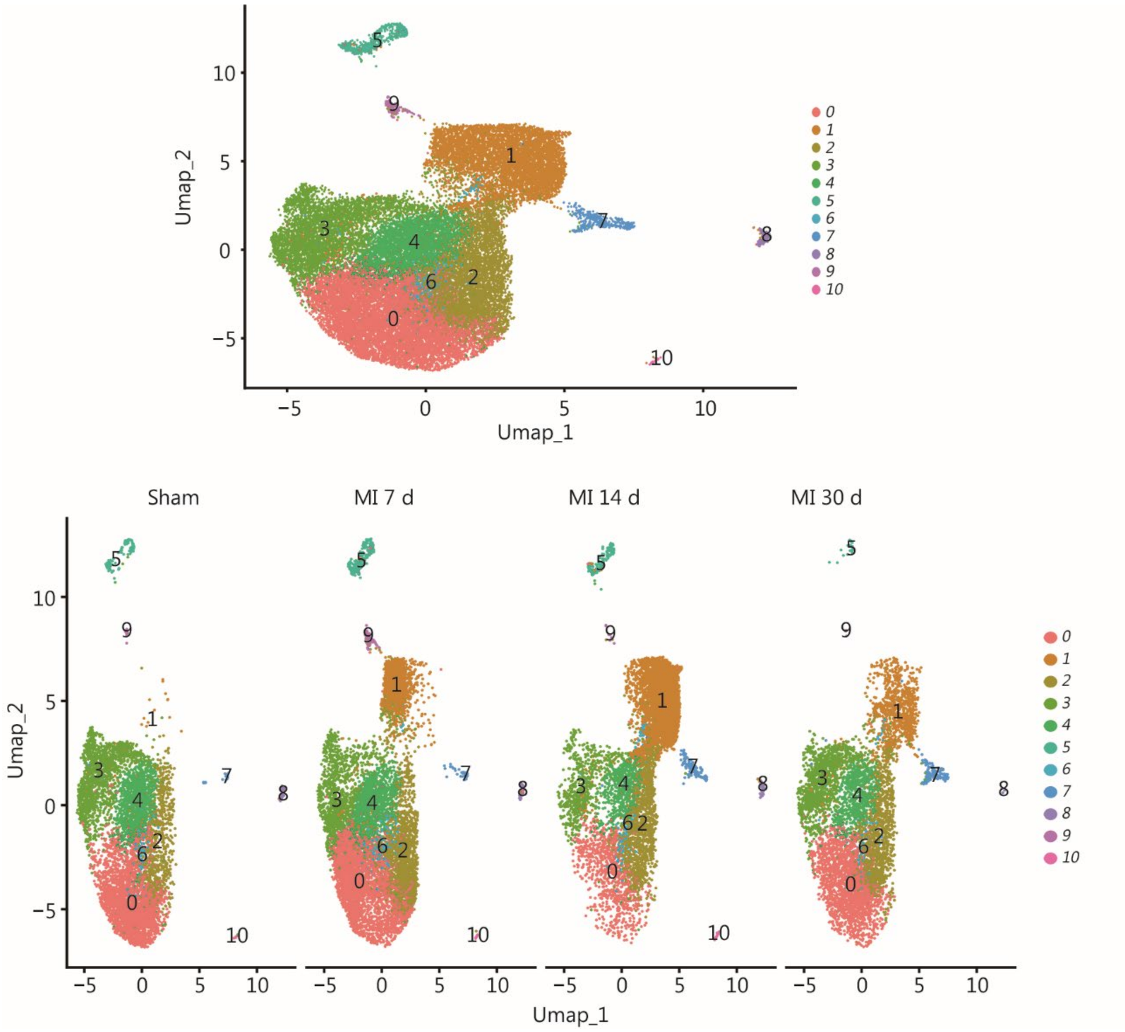

Fig. S2 Cardiac fibroblasts were divided into different subgroups and the changes of each subgroup after myocardial infarction were analyzed (Single cell database GSE132146). UMAP uniform manifold approximation and projection for dimension reduction, MI myocardial infarction

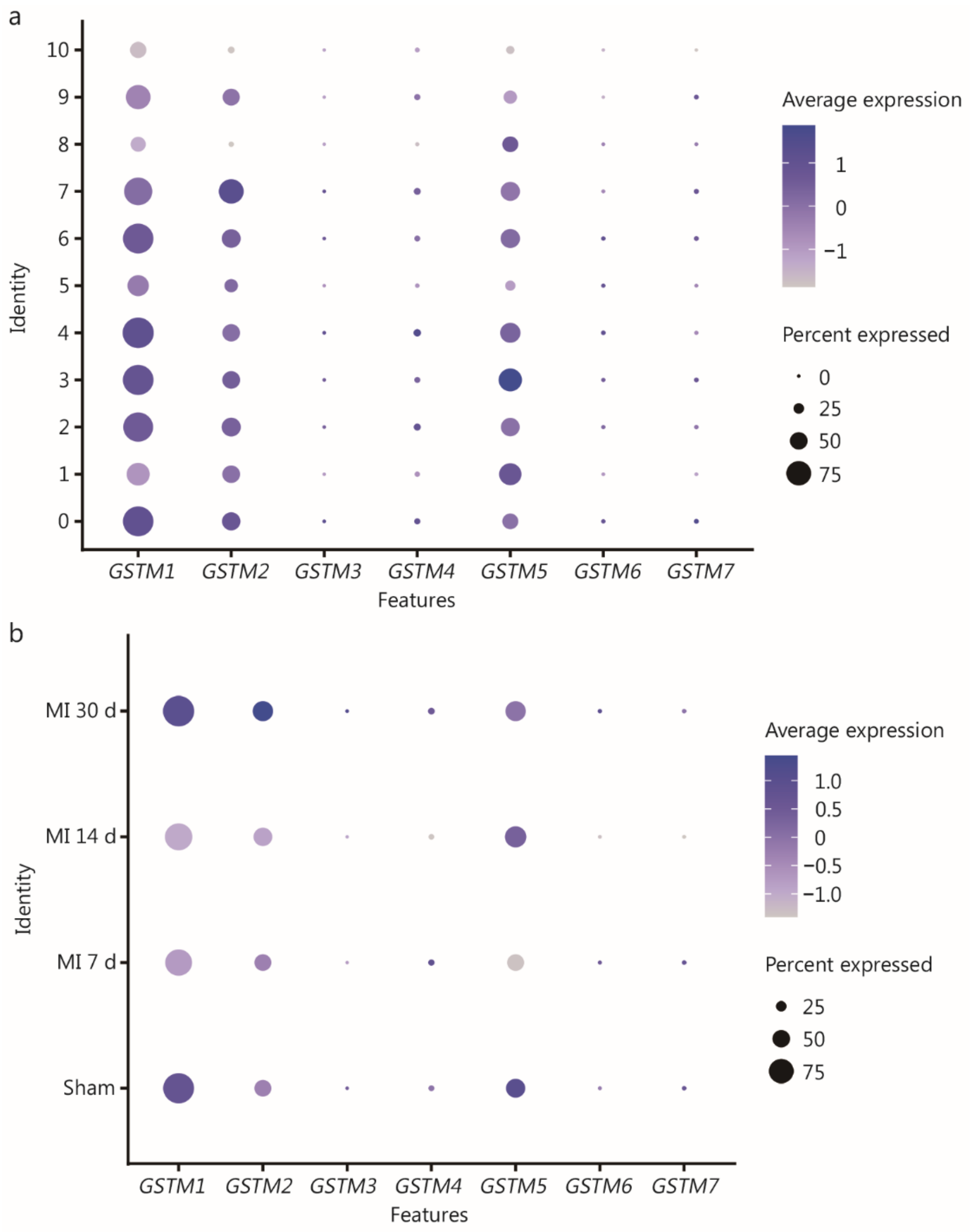

Fig. S3 Single-cell analysis (GSE 132146). **a** Basic expression of the GSTM family in different subgroups of mouse cardiac fibroblasts. **b** Expression of GSTM family in fibroblasts after MI. MI myocardial infarction, GSTM glutathione S-transferase mu

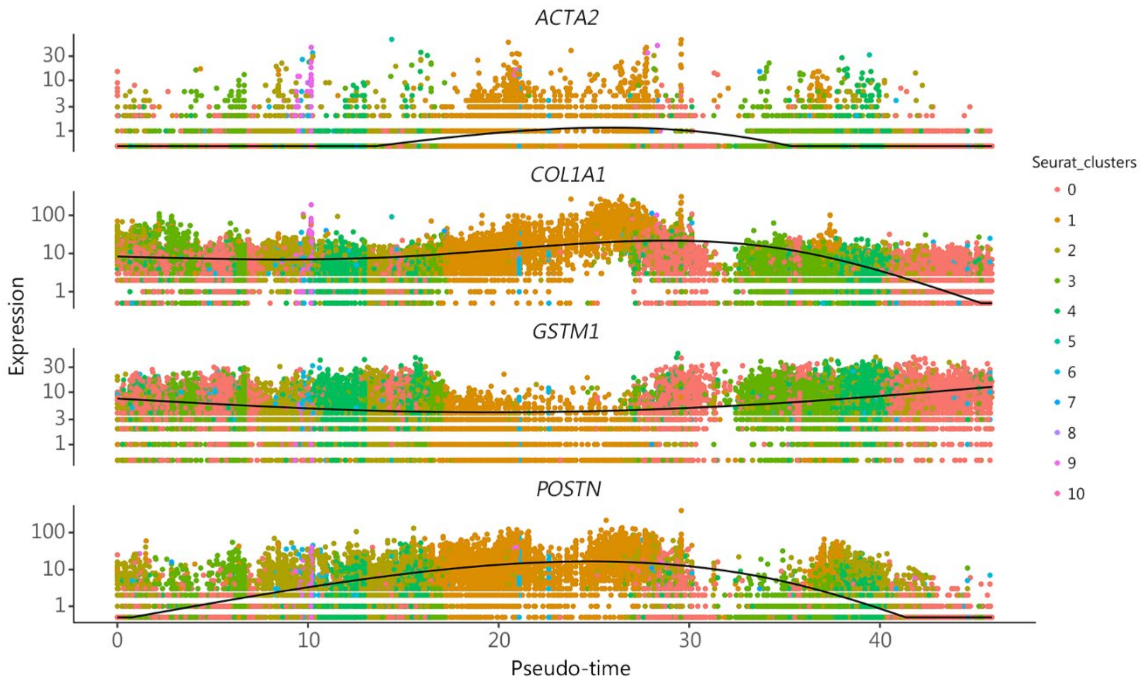

Fig. S4 Single-cell analysis (GSE 132146). GSTM1 and fibrosis-related indexes in post-MI fibroblasts were analyzed in pseudo-time. ACTA2 actin alpha 2, COL1A1 collagen type I alpha 1 chain, GSTM1 glutathione S-transferase mu 1, POSTN periostin

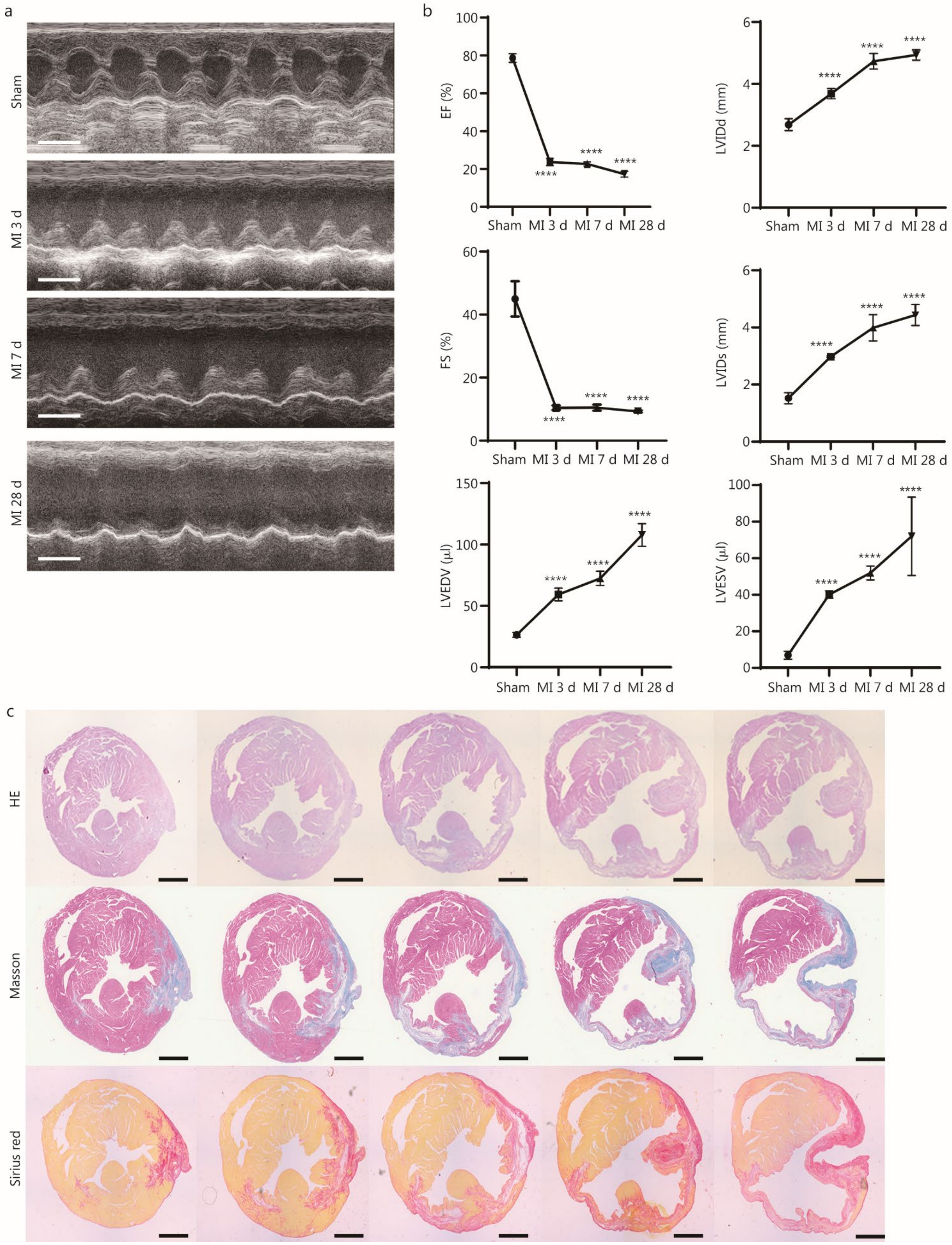

Fig. S5 Identification of myocardial infarction model. **a** Representative echocardiography of sham group of C57BL/6J mice and at day 3, 7, and 28 after myocardial infarction. Timestamp = 100 ms. **b** Ultrasound statistics of ventricular ejection fraction (EF), short-axis shortening rate (FS), left ventricular internal dimension at end-diastole

(LVIDd), left ventricular internal dimension at end-systole (LVIDs). Left ventricular end-diastolic volume (LVEDV), and left ventricular end-systolic volume (LVESV) in mice, with 6 biological repeats (n = 6). **c** HE, Masson and Sirius red stain of the heart sections of mice taken 28 d after myocardial infarction (Scale bar = 1 mm), with 6 biological repeats (n = 6). Statistical differences among three or more groups were conducted using one-way ANOVA. Data results are presented as mean ± standard error, $^{****}P < 0.0001$. MI myocardial infarction, HE Hematoxylin-Eosin

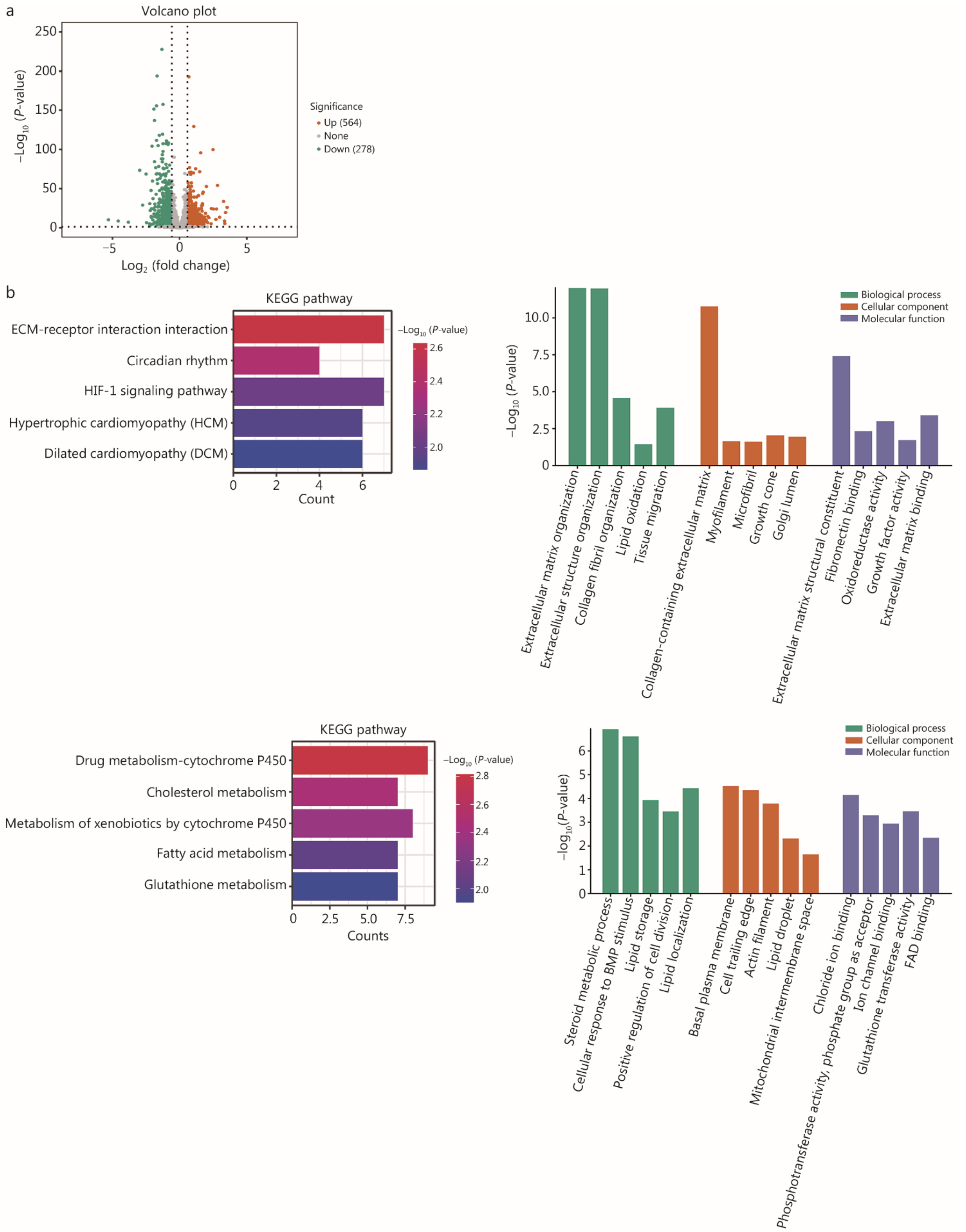

Fig. S6 Kyoto Encyclopedia of Genes and Genomes (KEGG) and Gene Ontology (GO) analysis of differentially expressed genes. **a** Volcano plot of differential genes obtained by RNA-seq analysis. **b** GO and KEGG enrichment analysis of up-regulated differential genes. **c** GO and KEGG enrichment analysis of down-regulated differential genes. FAD flavin adenine dinucleotide

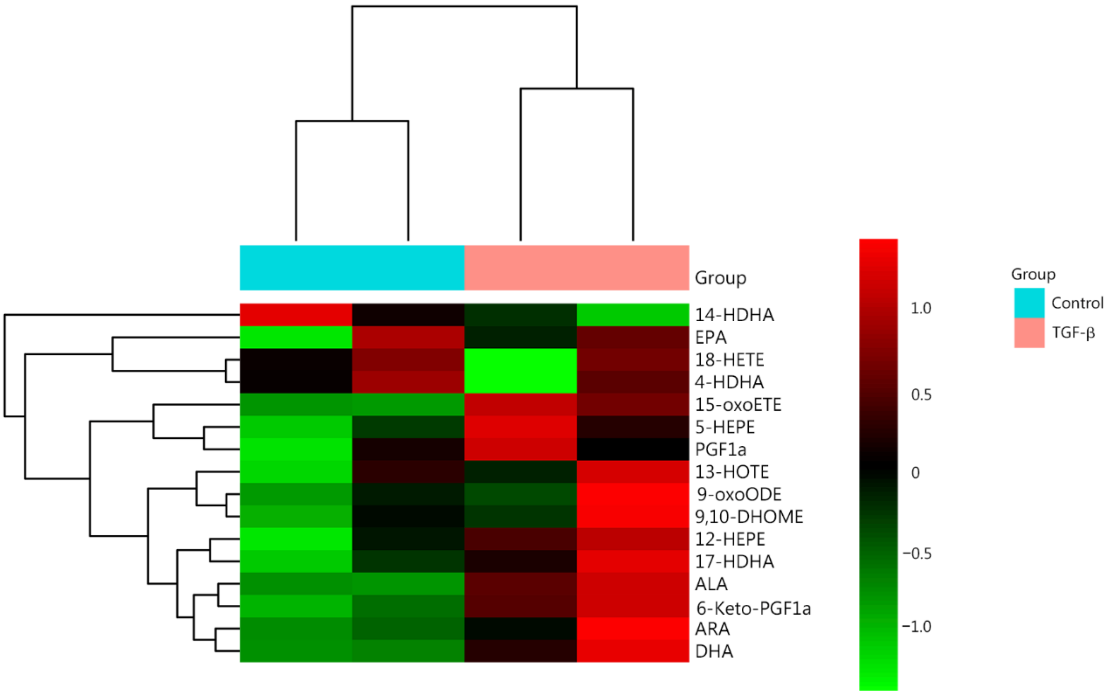

Fig. S7 Metabolite heat map. Adult mouse heart primary fibroblasts were isolated in vitro and stimulated with 10 ng/ml concentration of TGF-β for 48 h. The differential metabolite heat map of oxidative lipid metabolism between the control group and TGF-β group, with 2 biological repeats ($n = 2$). HDHA hydroxyacyl-CoA dehydrogenase, EPA eicosapentaenoic acid, HETE hydroxyeicosatetraenoic acid, HEPE hydroxyeicosapentaenoic acid, HOTE hydroxyoctadecatrienoic acid, ETE eicosatetraenoic acid, OXO oxoicosanoic, PG prostaglandin, ODE octadecadienoic acid, DHOME dihydroxy octadecenoic aci, ALA α-Linolenic acid, PGF1α prostaglandin F1α, ARA arachidonic acid, DHA docosahexaenoic acid, TGF-β transforming growth factor-β

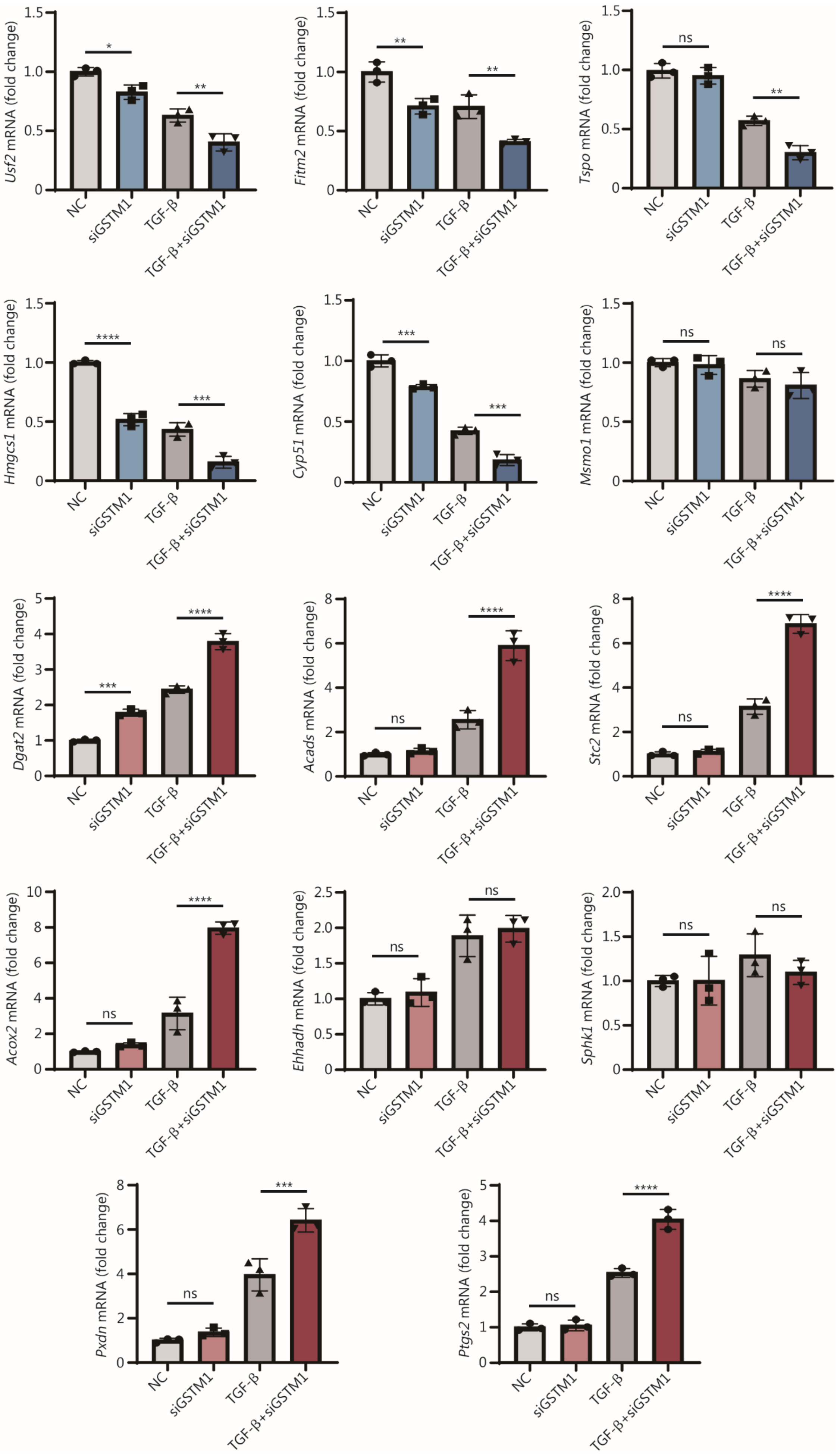
Usf2 mRNA (fold change)
Fitm2 mRNA (fold change)
Tspo mRNA (fold change)
Hmgcs1 mRNA (fold change)
Cyp51 mRNA (fold change)
Msmo1 mRNA (fold change)
Dgat2 mRNA (fold change)
Acads mRNA (fold change)
Stc2 mRNA (fold change)
Acox2 mRNA (fold change)
Ehhadh mRNA (fold change)
Sphk1 mRNA (fold change)
Pxdn mRNA (fold change)
Ptgs2 mRNA (fold change)
NC
siGSTM1
TGF-β
TGF-β+siGSTM1
ns
*
**
***
****

Fig. S8 PCR validation of differentially expressed genes associated with lipid peroxidation identified by RNA-seq. qPCR analysis of differentially expressed genes associated with lipid peroxidation mRNA levels in mouse fibroblasts stimulated by TGF-β for 48 h after siNC and siGSTM1 pretreatment, with 3 biological repeats (n = 3). Statistical differences among the four groups were conducted using one-way ANOVA. Data are expressed as mean ± standard error. ns non-significance, $^{*}P < 0.05$, $^{**}P < 0.01$, $^{***}P < 0.001$, $^{****}P < 0.0001$. NC negative control, Usf2 upstream transcription factor 2, Fitm2 fat storage inducing transmembrane protein 2, Tspo translocator protein, Hmgcs1 3-hydroxy-3-methylglutaryl-CoA synthase 1, Cyp51 cytochrome P450, family 51, Msmo1 methylsterol monooxygenase 1, Dgat2 diacylglycerol O-acyltransferase 2, Acads acyl-CoA dehydrogenase short chain, Stc2 stanniocalcin 2, Acox2 acyl-CoA oxidase 2, Ehhadh enoyl-CoA hydratase and 3-hydroxyacyl CoA dehydrogenase, Sphk1 sphingosine kinase 1, Pxdn peroxidasin, Ptgs2 prostaglandin-endoperoxide synthase 2

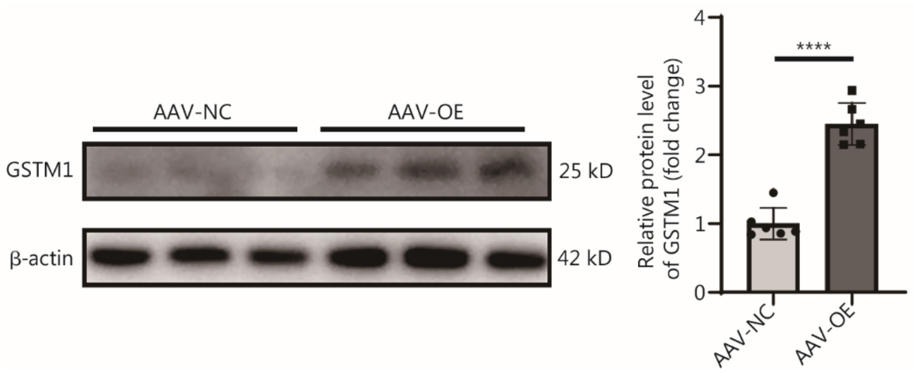

Fig. S9 Validation of AAV-mediated GSTM1 overexpression efficiency. Western blotting analysis and quantification of AAV-mediated GSTM1 overexpression in the heart with 6 biological repeats (*n* = 6). Data are expressed as mean ± standard error. $^{****}P < 0.0001$. AAV adeno-associated virus, OE overexpression, NC negative control, GSTM1 glutathione S-transferase mu 1

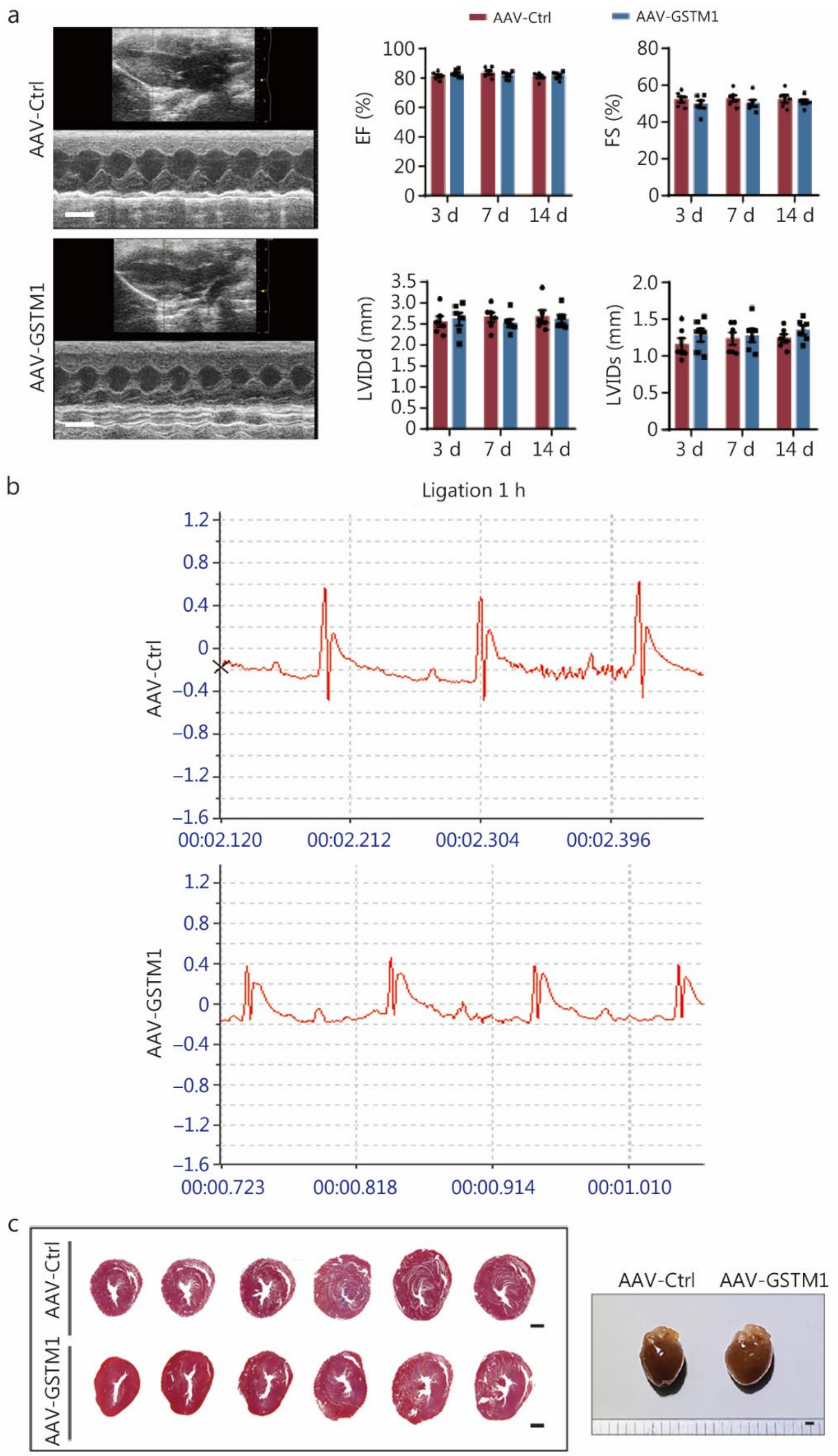

Fig. S10 Cardiac function, electrocardiogram, and cardiac morphology in GSTM1-AAV9 and GSTM1-Ctrl mice at baseline. **a** Representative map of basal cardiac function detected by echocardiography in mice with over-expression of GSTM1-AAV9 virus injected into tail vein compared with negative injection control. Statistical maps of ultrasonic ventricular ejection fraction (EF), fraction shorting (FS), left ventricular internal dimension at end-diastole (LVIDd), and left ventricular internal dimension at end-systole (LVIDs) in mice, with 6 biological repeats (n = 6). Timestamp

= 100 ms. **b** Electrocardiogram (ECG) of the control group and GSTM1 overexpressed group mice after myocardial infarction for 1 hour. **c** HE staining of the hearts of mice overexpressing GSTM1-AAV9 virus and negative control (Scale bar = 1 mm). Cardiogram of mice overexpressing GSTM1-AAV9 virus and negative control ((Scale bar = 1 mm). Statistical differences between the two groups were determined using the Student's *t*-test (a). Data results are presented as mean ± standard error. $^{*}P < 0.05$, $^{**}P < 0.01$, $^{***}P < 0.001$, $^{****}P < 0.0001$. Ctrl control, AAV adeno-associated virus, GSTM1 glutathione S-transferase mu 1

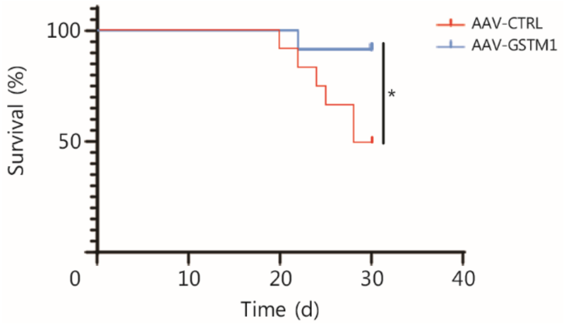

Fig. S11 Survival curve of mice after myocardial infarction

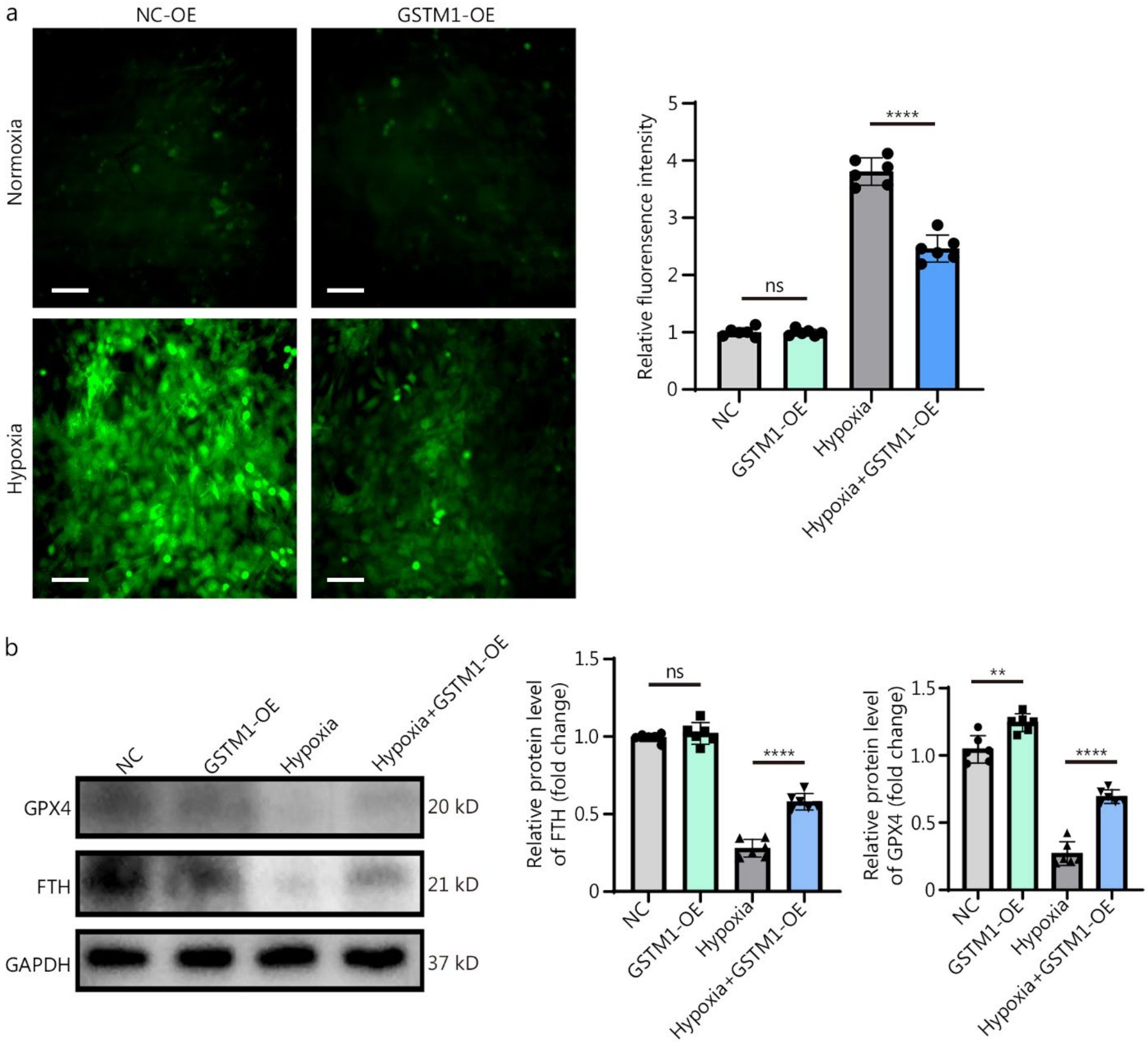

Fig. S12 Effects of GSTM1 on oxidative stress and ferroptosis in cardiomyocytes. **a** DCFH staining was used to compare the changes in ROS and the influence of GSTM1 overexpression on cardiomyocytes after hypoxia (Scale bar = 50 μm). **b** Western blotting detects the effect of GSTM1 on the ferroptosis of cardiomyocytes under hypoxia. ns non-significance, $^{**}P < 0.01$, $^{****}P < 0.0001$. ROS reactive oxygen species, OE overexpression, NC negative control, GSTM1 glutathione S-transferase mu 1, GPX4 glutathione peroxidase 4, FTH ferritin heavy chain, GAPDH glyceraldehyde-3-phosphate dehydrogenase

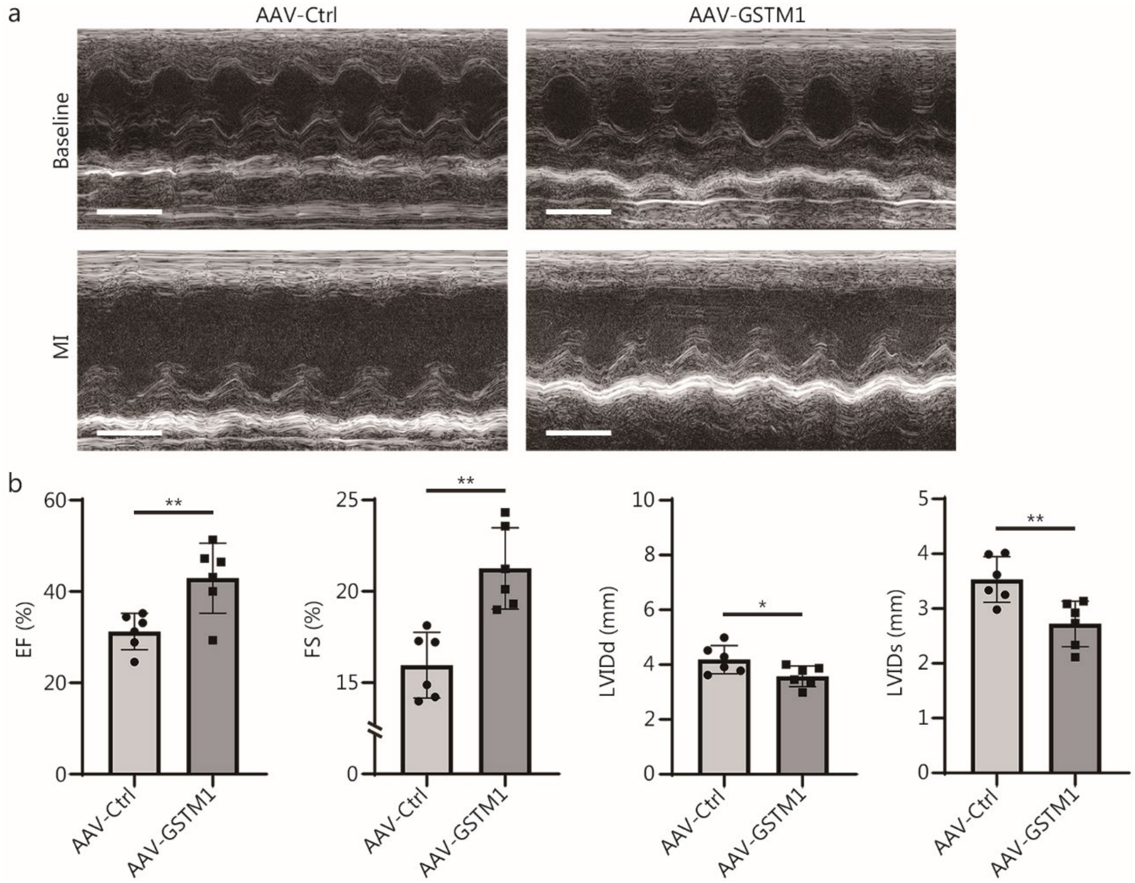

Fig. S13 AAV-mediated GSTM1 overexpression protects infarcted hearts in female mice. **a** Echocardiography at baseline and 14 d after myocardial infarction of female mice with over-expression of GSTM1-AAV9 virus injected in the tail vein compared with the negative injection control. Timestamp = 100 ms. **b** Echogram of mice ventricular ejection fraction (EF), Fraction shorting (FS), left ventricular internal dimension at end-diastole (LVIDd) and left ventricular internal dimension at end-systole (LVIDs) with 6 biological repeats (n = 6). Data are expressed as mean ± standard error. $^{*}P < 0.05$, $^{**}P < 0.01$, $^{***}P < 0.001$, $^{****}P < 0.0001$. AAV adeno-associated virus, MI myocardial infarction, Ctrl control, GSTM1 glutathione S-transferase mu 1

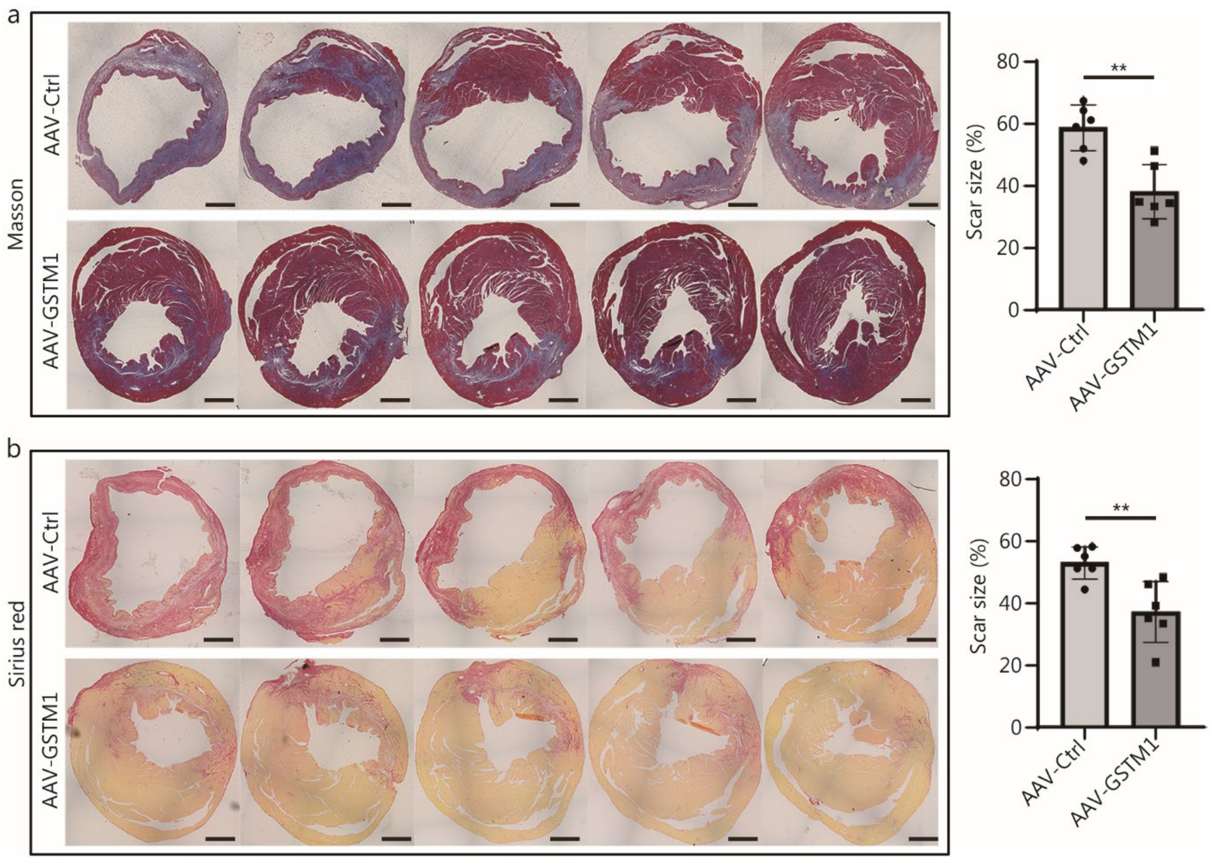

Fig. S14 AAV-mediated GSTM1 overexpression alleviated cardiac fibrosis after MI in female mice. Masson (**a**) and Sirius red (**b**) staining maps and statistical maps of infarct size were performed on the heart tissues of female mice 14 d after myocardial infarction with 6 biological repeats (n = 6) (Scale bar = 1 mm). Data are expressed as mean ± standard error. $^{*}P < 0.05$, $^{**}P < 0.01$, $^{***}P < 0.001$, $^{****}P < 0.0001$. AAV adeno-associated virus, Ctrl control, GSTM1 glutathione S-transferase mu 1
